# Plants respond to herbivory through sequential induction of cheaper defenses before more costly ones

**DOI:** 10.1371/journal.pbio.3003280

**Published:** 2025-08-14

**Authors:** Jinlong Wan, Jiahui Yi, Xiao Sun, Evan Siemann, Matthias Erb, Wei Huang

**Affiliations:** 1 Wuhan Botanical Garden, Chinese Academy of Sciences, Wuhan, China; 2 Hubei Key Laboratory of Wetland Evolution & Ecological Restoration, Wuhan Botanical Garden, Chinese Academy of Sciences, Wuhan, China; 3 University of Chinese Academy of Sciences, Beijing, China; 4 School of Life Sciences, Henan University, Kaifeng, China; 5 Department of Biosciences, Rice University, Houston, Texas, United States of America; 6 Institute of Plant Sciences, University of Bern, Bern, Switzerland; Cornell University, UNITED STATES OF AMERICA

## Abstract

Plants encounter natural antagonist threats of varying intensity and respond by activating multiple defense traits. Due to the fitness costs associated with producing defense traits, plants are expected to activate less costly traits first, reserving more costly defenses for potentially more severe damage (“cheaper first hypothesis”), but evidence to date is scarce. Here, we tested this hypothesis by measuring six putative defense traits in the annual plant *Ambrosia artemisiifolia*. We found that all traits were effective against insect herbivores, but production of three of them more strongly reduced plant growth, suggesting higher growth costs. When plants were attacked by insect herbivores, less costly traits were induced first, even at the lowest levels of damage, while more costly traits were activated only after higher damage thresholds. This cost-dependent sequential pattern was consistently observed in plants when challenged by 12 different herbivore species from three insect orders. These findings demonstrate that plants can employ the “cheaper first” sequential induction defense strategy, potentially allowing them to reduce defense costs and maximize fitness. Our study provides new insights into how plants fine-tune their defense responses under variable antagonistic pressures.

## Abstract 2

Plants encounter natural threats of varying intensity and respond by activating multiple defense traits. This study of six defense traits in Ambrosia artemisiifolia reveals that when attacked by insect herbivores, less costly traits are induced first, while more costly traits are activated only after higher damage thresholds.

## Introduction

Plants often experience varying levels of attack from herbivores and pathogens. To counteract these threats, they have developed various mechanical (*e.g.*, spines and trichomes) and chemical defense traits (*e.g.*, phenolics and terpenoids) [1,2]. However, because the production or expression of defense traits incurs fitness costs [3–5], selection should favor resource allocation to defenses that minimize costs while providing sufficient protection from antagonists (optimal defense theory) [6,7]. Defensive traits can be expressed constitutively or induced in response to damage, which alters defense gene expression through hormonal signaling pathways (*e.g.*, jasmonic acid) [8–11]. Compared with constitutive defenses, induced defenses are considered to be a resource-saving strategy because they allow plants to only invest in defenses when necessary and avoid costs when natural antagonists are absent [12,13]. As damage levels increase, induced defenses typically exhibit either continuous or threshold reaction norms, with the specific level of damage required to trigger induction primarily depending on the costs of defensive traits [14]. Because costs vary among defense traits [15], it has been predicted that lower-cost defense traits will be induced first (*i.e.*, by mild damage) while increasingly higher-cost defense traits will only be deployed when the protection provided by lower-cost defenses is insufficient under more severe damage [16,17]. Here, we refer to this as the “cheaper first hypothesis.”

Several studies have shown that plant defense traits are induced asynchronously along damage intensity gradients [18–21]. For example, the toxic chemical hydrogen cyanide increased consistently in *Acacia sieberiana* with increasing browsing damage, whereas spines, a mechanical defense, only increased in length when damage exceeded a relatively higher level [18]. Similarly, latex, a sticky emulsion, and lignin, a phenolic polymer, in *Asclepias syriaca* exhibited sequential induction when leaf damage intensified [20]. However, few studies have tested whether such induction sequences reflect the relative costs of the traits involved. To the best of our knowledge, empirical support for this “cheaper first hypothesis” that predicts lower-cost defenses are triggered before higher-cost ones, comes almost entirely from animal systems [22–24]. Unlike animals, which often coordinate induced defenses through centralized systems, plant defense responses tend to be more decentralized [25,26]. This fundamental difference in defense mechanisms raise uncertainty about whether plants can employ a “cheaper first” sequential induction strategy, which could have important implications for our understanding of plant defense strategies against their natural antagonists.

Here, we tested the “cheaper first hypothesis” using the annual plant species, common ragweed (*Ambrosia artemisiifolia*, Asteraceae) and 12 generalist insect herbivore species from three orders (Coleoptera, Lepidoptera, and Orthoptera). By focusing on six putative defense traits, chlorogenic acid (hereafter CHA), kaempferol, rutin, condensed tannins (hereafter tannins), lignin, and trichomes, we examined their defense efficacy, fitness costs (in terms of plant growth), and induction sequence in response to increasing levels of herbivory. We predict the following: (1) all six traits are effective defenses against generalist insect herbivores of common ragweed; (2) the production of less-recyclable structural defenses (trichomes) and large-molecule polymeric compounds (tannins, lignin) will impose higher growth costs than small-molecule metabolites (CHA, kaempferol, rutin); and (3) less costly traits will be induced at lower levels of insect herbivore damage than more costly traits.

## Results

### Effects of six putative defense traits on herbivores

To examine the effects of six putative defense traits against common ragweed herbivores, we first conducted growth chamber experiments using common ragweed plants grown from seeds collected from 12 natural populations around Wuhan, China (S1A Fig; Table A in S1 File). For each plant, we measured the levels of the six traits and the performance of herbivores feeding on the leaves. We used three naturally occurring herbivores, *Spodoptera litura* (Lepidoptera: Noctuidae), *Monolepta hieroglyphica* (Coleoptera: Chrysomelidae), and *Atractomorpha sinensis* (Orthoptera: Pyrgomorphidae) (Tables B and C in S1 File), to conduct the feeding bioassays, resulting in three independent experiments (12 populations × 6 replicates = 72 plants per herbivore species). For each herbivore species, we quantified defense efficacy of traits by analyzing how herbivore performance depended on trait expression levels across different plant individuals (*n* = 66 for *S. litura* due to six larval deaths).

**Fig 1:**
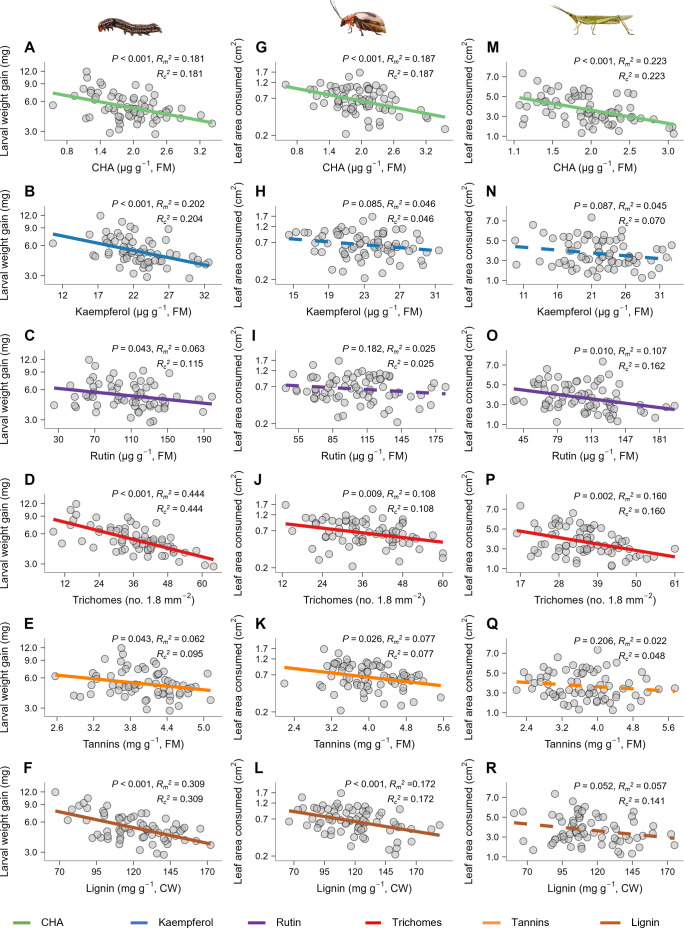
Six traits contribute to defense against multiple types of herbivores on *Ambrosia artemisiifolia.* Relationships between levels of each of six traits, chlorogenic acid (CHA), kaempferol, rutin, trichomes, condensed tannins (tannins) and lignin, and the performance of each of three herbivores, including *Spodoptera litura* **(A-F)**, *Monolepta hieroglyphica* **(G-L)**, and *Atractomorpha sinensis* **(M-R)**. Larval weight gain was measured for *S. litura*, and leaf area consumed was measured for *M. hieroglyphica* and *A. sinensis*. For each herbivore species, each plant trait was analyzed separately using a linear mixed model across plants from 12 *A. artemisiifolia* populations, with herbivore performance as the response variable and trait levels as the fixed factor along with population as a random factor. In each subplot, data points represent individual replicates (n = 6 per plant population). The different line colors represent different traits. Solid lines indicate significant relationships (Benjamini–Hochberg adjusted **p* *< 0.05) between defense traits and herbivore performance while dotted lines indicate nonsignificant relationships between them (Benjamini–Hochberg adjusted **p* *≥ 0.05). Adjusted *p*-values, marginal *R*^2^ and conditional *R*^2^ are given. Note the log scale of larval weight gain of *S. litura* **(A-F)** and leaf area consumed by *M. hieroglyphica* **(G-L)** on the y-axis. FM, leaf fresh biomass. CW, cell wall. The data underlying this figure can be found in https://doi.org/10.6084/m9.figshare.29364695.

When each trait was analyzed individually in a linear mixed model (LMM), with that trait as the sole fixed factor and population as a random factor, we found that all traits negatively affected herbivore performance (*i.e.*, the larval growth of *S. litura* and leaf area consumed by adult *M. hieroglyphica* and adult *A. sinensis*), although these effects were not always consistent across all herbivore species (Fig 1; Table D in S1 File). For example, CHA significantly negatively affected all three herbivores (Fig 1A, 1G and 1M; Table D in S1 File) while kaempferol negatively affected only *S. litura* (Fig 1B; Table D in S1 File). For each herbivore species, pairwise correlation analyses among the six traits showed no significant correlations between any pair of traits (Table E in S1 File). We then performed an LMM for each herbivore, including all six traits as fixed factors (population still as a random factor), and found that most negative effects persisted after accounting for the influence of other traits (Table F in S1 File). Importantly, each trait retained a significant negative effect on at least one herbivore species (Table F in S1 File). Together, these results suggest that each trait independently serves as an effective defense against herbivores.

### Costs of defense traits in terms of plant growth

To evaluate whether the production of trichomes, tannins, and lignin incurred higher growth costs than that of CHA, kaempferol, and rutin, we conducted a common garden experiment using plants from the above 12 common ragweed populations (100 plants per population; S1B Fig; Table A in S1 File). For each plant, we measured the levels of the six traits and plant biomass. We quantified the costs of these traits by analyzing how plant biomass depended on trait expression levels across different plant individuals.

**Fig 2:**
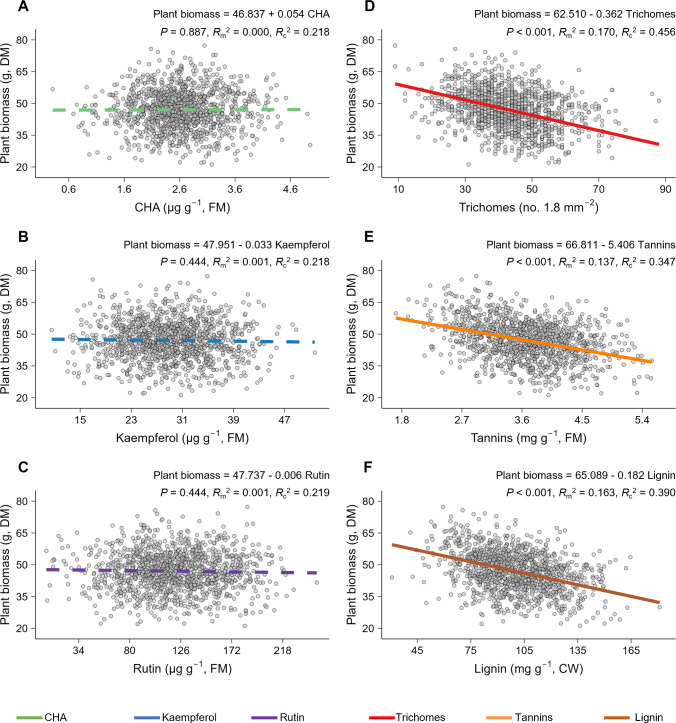
The production of six defense traits incurs varying growth costs in *Ambrosia artemisiifolia.* Relationships between levels of each defense trait, chlorogenic acid (CHA; **A**), kaempferol (**B**), rutin (**C**), trichomes (**D**), condensed tannins (tannins; **E**) and lignin (**F**) and plant biomass based on 12 natural *A. artemisiifolia* populations. Each trait was analyzed separately using a linear mixed model with plant biomass as the response variable and trait levels as the fixed factor along with population as a random factor. Data points represent individual replicates (n = 100 per plant population). Solid lines indicate significant relationships (Benjamini–Hochberg adjusted **p* *< 0.05) between defense traits and plant biomass while dotted lines indicate nonsignificant relationships between them (Benjamini–Hochberg adjusted *p* ≥ 0.05). The different line colors represent different traits. Coefficients for the fixed factors, adjusted *p*-values, marginal *R*^2^ and conditional *R*^2^ are given. FM, leaf fresh biomass; DM, plant dry biomass. CW, cell wall. The data underlying this figure can be found in https://doi.org/10.6084/m9.figshare.29364695.

When each trait was analyzed individually in an LMM, with that trait as the sole fixed factor and population as a random factor, we found no significant relationship between the concentrations of CHA, kaempferol or rutin, and plant biomass (Fig 2A-C; Table G in S1 File). In contrast, increasing levels of trichomes, tannins, and lignin each significantly reduced plant growth (Fig 2D-F; Table G in S1 File). For example, an approximately 9-fold increase in trichome densities resulted in a nearly 50% decrease in plant biomass (Fig 2D). Of the 15 pairwise correlations among the six traits, only three trait pairs (trichomes–tannins, trichomes–lignin, and tannins–lignin) were significantly positively correlated, whereas none of the other 12 pairs showed significant correlations (Table H in S1 File). We then conducted an LMM that included all six traits as fixed factors (population still as a random factor) and found that the observed effect for each trait individually was still present after accounting for the influence of other traits (Table I in S1 File). By pairwise comparisons of regression coefficients within the LMM with multiple fixed factors, we found that trichomes, tannins, and lignin, indeed, had stronger negative effects compared to the other three traits (Table J in S1 File). These results indicate that the production of trichomes, tannins, and lignin incurs higher growth costs than that of CHA, rutin, and kaempferol.

### Induction sequence of defense traits with increasing damage

To test the induction sequence of defense traits with varying costs in response to increasing damage, we conducted growth chamber experiments with 12 generalist chewing insect herbivores (S1C Fig; Table C in S1 File). For each herbivore species separately, we manipulated its density to cause varying amounts of leaf area removal on individual plants (seven densities per species, with 18 replicates per density; Table C in S1 File). Then, we measured the levels of the six defense traits in these plants. Due to limitations in insect availability and experimental feasibility, we used plants from one common ragweed population (HP3; Table A in S1 File). This population was chosen because its growth-cost patterns (*i.e.*, significant plant biomass reductions by tannin, lignin, and trichome production but not by CHA, kaempferol, or rutin production) matched those observed in a majority of populations (7 of 12; Table K in S1 File) and aligned with the overall pattern seen when data from all 12 populations were combined (Fig 2; Tables G and I in S1 File). For each herbivore species, we analyzed each trait using both linear and segmented models to estimate the relationship between trait levels and the amount of leaf damage and selected the best-fitting model to describe its reaction norm (linear or segmented). We determined the induction sequence of traits by comparing their induction thresholds as damage increased.

**Fig 3:**
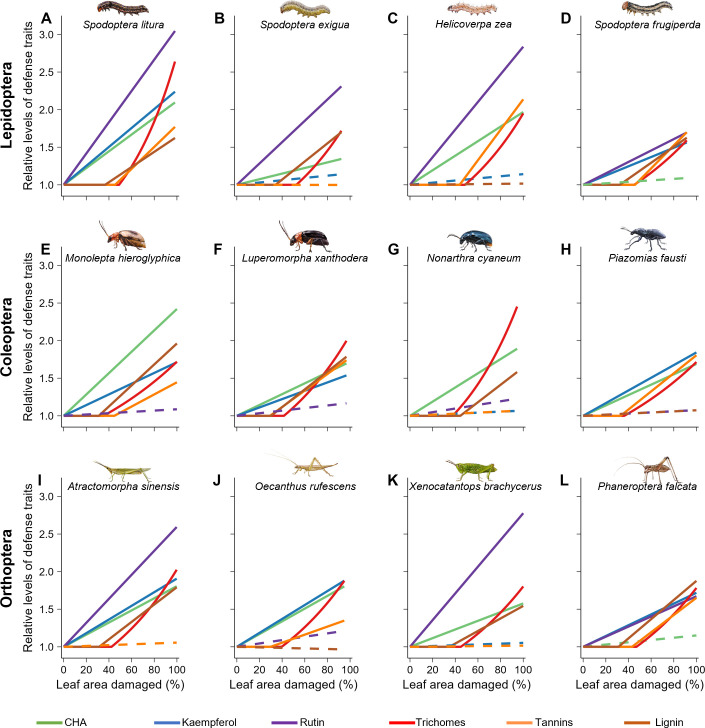
Lower-cost traits are induced at lower levels of herbivory than higher-cost traits in *Ambrosia artemisiifolia.* Relationships between levels of each defense trait, chlorogenic acid (CHA), kaempferol, rutin, trichomes, condensed tannins (tannins), and lignin, and the percentage of leaf area damaged by each of 12 herbivores. For each herbivore treatment, the six traits were analyzed separately. The reaction norm for each trait was chosen between a linear or segmented model based on a combination of the Davies test and AIC/QAIC. The relative change in trait values was calculated by dividing the predicted values of each trait by the model’s y-intercept. The different line colors represent different traits. Solid lines indicate significant effects (adjusted 95% confidence intervals not crossing zero) of herbivore damage on levels of traits while dotted lines indicate nonsignificant effects (adjusted 95% confidence intervals crossing zero). Statistical results are in Table L in S1 File. More detailed plots based on the original models are in S2-S13 Figs. The data underlying this figure can be found in https://doi.org/10.6084/m9.figshare.29364695.

Overall, regardless of the reaction norms, each trait was increasingly induced as damage levels increased; however, not all traits were induced by every herbivore (Figs 3 and S2-S13; Table L in S1 File). Specifically, only trichomes were significantly induced by all 12 herbivore species (see the red lines in Figs 3 and S2-S13), while the other traits were induced by only a subset of herbivores. For example, rutin was significantly induced by all Lepidoptera (purple lines in Figs 3A-D and S2-S5) and most Orthoptera (purple lines in Figs 3I, 3K, 3L, S10, S12, and S13) but not by any Coleoptera. Likewise, tannins were induced by most Lepidoptera (orange lines in Figs 3A, 3C, 3D, S2, S4, and S5) and Coleoptera (orange lines in Figs 3E, 3F, 3H, S6, S7, and S9) but were less likely to be induced by Orthoptera (orange lines in Figs 3J, 3L, S11, and S13).

For each trait, when it was significantly induced, the shape of reaction norm (*i.e.*, linear versus segmented) showed high consistency across different herbivores. Specifically, the effects of damage on concentrations of CHA, kaempferol, and rutin were best explained by a linear model (Table L in S1 File). The concentrations of CHA, kaempferol, and rutin increased at the lowest levels of leaf area removal (just above 0%) and continued to increase with higher amounts of damage (see the green, blue, and purple lines in Figs 3 and S2-S13). In contrast to these three traits, effects of damage on levels of trichomes, tannins, and lignin were best explained by a segmented model with a single breakpoint for each herbivore (Table L in S1 File). The levels of trichomes, tannins, and lignin did not change until the amount of leaf area removed increased above a certain threshold (29.4% to 52.5% removal) and then consistently increased afterwards (see the red, orange, and brown lines in Figs 3 and S2-S13). These results suggest that CHA, kaempferol, and rutin are induced at lower levels of herbivory intensity compared to trichomes, tannins, and lignin.

## Discussion

In this study, we measured six putative defense traits, CHA, kaempferol, rutin, tannins, lignin, and trichomes, in the annual plant, common ragweed, to examine their effects on herbivore performance, fitness costs (in terms of plant growth), and induction sequence in response to increasing levels of herbivory. We showed that all six traits were effective defenses against herbivores, with trichomes, tannins, and lignin showing stronger negative correlations with plant biomass production, suggesting that production of these three traits incurred higher growth costs. More importantly, less costly traits (CHA, kaempferol, and rutin) were induced prior to more expensive ones as herbivory levels increased, and this cost-dependent sequential pattern was consistent in plants when fed upon by a variety of herbivore species. These findings provide clear empirical evidence that plants can employ a “cheaper first” sequential induction strategy to defend against their natural antagonists.

In addition to relative costs, other factors may contribute to the observed sequential pattern between the induction of CHA, kaempferol, and rutin and that of trichomes, tannins, and lignin. First, the accumulation of large polymers (*e.g.*, tannins and lignin) and macroscopic structures (*e.g.*, trichomes) tend to require more time to reach sufficient levels, potentially resulting in more delayed responses compared to small-molecule chemicals [27]. However, our experimental design, which examined induced responses in plants exposed to varying levels of damage over the same time period, allowed us to rule out this possible explanation. Second, for traits with similar costs, those that are more effective defenses may be induced at lower levels of damage [14,28]. Therefore, the earlier induction of CHA, kaempferol, and rutin as damage increases may reflect higher defense efficacy. To assess this possibility, we first identified which traits were significantly induced by each of three herbivores (*S. litura*, *M. hieroglyphica*, and *A. sinensis*) in Experiment 3. We then used the corresponding data from Experiment 1 to compare the defense efficacy of those induced traits against the same herbivore species. We found that CHA, kaempferol, and rutin, that were induced earlier by *A. sinensis*, tended to show higher defense efficacy against this insect than later induced trichomes and lignin (*p* = 0.054; Table M in S1 File), suggesting that efficacy may play a role in determining the induction order of defense traits. However, because this pattern was weak (marginally significant) and was not observed for *S. litura* or *M. hieroglyphica*, defense efficacy alone cannot likely explain the observed overall sequence pattern. The weak evidence for induction speed and defense efficacy as primary drivers points to a critical role of costs in this study.

Beyond cost-based prioritization, our results reveal trait induction is also shaped by herbivore identity. For example, rutin was strongly induced by all Lepidoptera and most Orthoptera but not by any Coleoptera, while tannins were induced by most Lepidoptera and Coleoptera, but rarely by Orthoptera (see Results). Coupled with the findings from Experiment 1, where rutin and tannins lacked defense efficacy against *M. hieroglyphica* (Coleoptera) and *A. sinensis* (Orthoptera), respectively, these results suggest that common ragweed may be able to identify the attacking herbivores and selectively activate traits that are effective against the attacker. This herbivore-specific induction of effective defenses has also been documented in other systems and may enable plants to avoid production of ineffective defenses [29–32].

Plants can detect highly specific herbivore-associated cues (*e.g.*, saliva, frass, and odor as well as feeding behavior and damage pattern) and initiate a hormone-mediated signaling cascade, ultimately leading to the expression of specific defense traits [32–34]. Therefore, the herbivore-specific trait induction observed here may reflect differences in cues from different herbivores. Despite this specificity, the consistent earlier induction of lower-cost traits across herbivores may correspond to the activation of corresponding signaling cascades at lower levels of damage. Mapping these perception-to-expression pathways in common ragweed would help to clarify the molecular mechanisms underlying the herbivore-specific, “cheaper first” sequential induction of defense traits.

The optimal defense theory assumes that plants evolve to allocate resources to defense by optimizing the cost–benefit ratio in terms of fitness [35]. Induced defense has long been considered to support the optimal defense theory because, compared with constitutive defense, it enables plants to reduce defense costs by avoiding unnecessary production of defense traits when herbivores are absent [36,37]. Here, we show that, even for induced defenses, plants can employ a “cheaper first” strategy to minimize the production of higher-cost defense traits at lower levels of damage. Such fine-tuning may allow plants to further reduce defense costs, thereby maximizing fitness. Therefore, our findings not only support the optimal defense theory but also expand upon it by providing a more nuanced understanding of how plants refine their defense responses based on both the costs of defense traits and the damage level they experience. In natural environments, herbivore pressure often exhibits significant spatiotemporal fluctuations [38]. Through this “cheaper first” strategy, plants can conserve resources during low herbivore pressure, while still maintaining the flexibility to activate more costly defenses when herbivore pressure intensifies. This dynamic adjustment may allow plants to effectively balance defense costs with fitness benefits, thereby providing a significant evolutionary advantage.

It should be noted that our study had limitations. First, using plant biomass alone (without reproductive measures) as a fitness proxy in Experiment 2 may limit the comprehensiveness of our assessment. However, plant growth and reproduction are often positively correlated [39], a relationship also shown in common ragweed [40]. Thus, biomass is likely a valid surrogate for fitness in this study. Second, induced defense incurs various resource costs, including production costs (resources required to generate traits) and maintenance costs (resources invested in maintaining the sensory and regulatory machinery of induced responses) [27,41,42]. In Experiment 2, we assessed the costs of defense traits by correlating their constitutive levels and growth of individual plants. This method reflects only phenotypic (not genetically based) production costs and does not capture the full spectrum of costs associated with induced defense. However, high production costs often parallel high maintenance costs, as large polymers (*e.g.*, lignin) typically require more complex regulatory mechanisms for their activation [43]. Therefore, production costs likely serve as a reliable proxy for overall resource costs. Third, unlike Experiments 1 and 2, Experiment 3 included only a single plant population which may constrain the generalizability of the findings. Although this population had defense–cost patterns similar to those of a majority of surveyed populations, future experiments that include multiple plant populations or a breeding design would provide a more rigorous test of the “cheaper first hypothesis.”

In conclusion, this study shows that plants can employ a “cheaper first” sequential induction strategy to defend against their natural antagonists. This finding not only supports but also expands the optimal defense theory, providing new insights into how plants optimize their defense strategies under highly variable antagonistic pressure. Although plants and animals differ fundamentally in their defense mechanisms [44], both employ this “cheaper first” strategy, suggesting that it may be a general defense pattern across diverse kingdoms.

## Materials and methods

### Study system

#### Plant material.

*A. artemisiifolia* (Asterales: Asteraceae), known as common ragweed, is an annual herbaceous plant native to North America that commonly occurs in disturbed habitats such as roadsides, abandoned fields, and agricultural areas throughout China [45]. It germinates in late March through April and flowers from mid-July through August at the study site, Wuhan, in central China. We collected seeds of common ragweed from 50 plants spaced at least 10 m apart in 12 populations with a minimum distance of 10 km between adjacent populations in rural areas of Wuhan (Table A in S1 File) in September 2021. We placed seeds of each plant in Petri dishes with moist sand in a refrigerator at 4°C for 3 months to break dormancy and transferred them to a growth chamber (14 h light/10 h dark with 24/18 °C temperature, relative humidity 50%–70%) for 5 days to initiate germination. Then, we transplanted seedlings into 72-cell seeding trays filled with peat-based seedling substrate (Klasmann-Deilmann GmbH, Germany). After 10 days of growth, we selected similar-sized seedlings with two pairs of true leaves for experiments.

#### Trait selection.

We focused on six putative defense traits of common ragweed, CHA, kaempferol, rutin, condensed tannins (tannins), lignin, and trichomes, based on the following criteria: (1) These traits have been previously identified in this plant species [46,47]. (2) They have been widely reported to be effective against generalist insect herbivores. Specifically, trichomes mainly act as a mechanical barrier that can interfere with the movement, feeding, and oviposition of herbivores [48]; lignin can inhibit the feeding by herbivores by increasing leaf toughness and reducing digestibility [48]; in addition to feeding deterrent and digestibility-reducing effects, the four secondary metabolites (*i.e*., tannins, CHA, rutin, and kaempferol) may also play a resistance role by toxicity [49–54]. (3) These traits have been reported to be inducible in various plant–herbivore systems [48,55,56]. (4) They are expected to exhibit significant variation in their associated costs because the production of less-recyclable physical defenses (*e.g.*, trichomes) and large-molecule defense chemicals (*e.g.*, tannins, lignin) has been hypothesized to impose higher fitness costs than production of small-molecule defense chemicals such as CHA, kaempferol, and rutin [57,58], although the generality of this pattern remains debated [15].

#### Herbivore assemblage.

A previous field survey that we conducted revealed that during the growing season, common ragweed is attacked by a large number of herbivores, including grasshoppers, beetles, and caterpillars, around the study site (see Text A and Table B in S1 File for more details on methods and results). This herbivore composition is similar to that observed in its native range [59]. In this study, we selected 12 chewing herbivores according to their availability to conduct experiments (Table C in S1 File). All species are generalist (polyphagous) herbivorous species (Table B in S1 File) [60,61]. Among these species, 10 species commonly occur on common ragweed in the fields around Wuhan Botanical Garden (located in Wuhan), including two Lepidoptera (*Helicoverpa armigera* and *S. litura*)*,* four Coleoptera (*M. hieroglyphica*, *Luperomorpha xanthodera*, *Nonarthra cyaneum*, and *Piazomias fausti*), and four Orthoptera (*A. sinensis*, *Oecanthus rufescens*, *Xenocatantops brachycerus*, and *Phaneroptera falcata*; Table B in S1 File). The remaining two species (*Spodoptera exigua* and *Spodoptera frugiperda*) were not observed on common ragweed in the field but are widely used in laboratory herbivory studies due to their broad host ranges [60,61]. All 12 species were used for the subsequent herbivory induction treatments and three of them, *S. litura*, *M. hieroglyphica*, and *A. sinensis*, were used for subsequent insect bioassays. The sources and developmental stages of these insects are provided in Table C in S1 File. Specialist herbivores were not included in this study due to the fact that some defense traits, especially CHA produced by common ragweed, stimulate host finding, feeding behavior, and oviposition of specialists, rather than serving as a defense trait [46,62,63].

### Experiment 1: effects of six putative defense traits on herbivores

To examine the defense efficacy of six putative defense traits (CHA, kaempferol, rutin, trichomes, tannins, and lignin) against herbivores in common ragweed, we conducted growth chamber experiments under the conditions described above in August 2022 (S1A Fig). We used plants from six maternal lines randomly selected within each of 12 plant populations (Table A in S1 File). From each maternal line, we randomly selected three seedlings and individually transplanted them into 1 L pots (12 cm in height × 11 cm in diameter) filled with a homogenized 50:50 mixture of seedling substrate and sand. After growth of 20 days, one plant from each maternal line was randomly assigned to each of three naturally occurring herbivore species, *S. litura*, *M. hieroglyphica*, and *A. sinensis* (3 herbivore species × 12 plant populations × 6 replicates = 216 plants; Table B in S1 File). For each plant, we used its sixth pair of leaves which had just fully expanded to conduct experiments: one leaf was used to measure six putative defense traits, and the other one was used to measure resistance to herbivory.

For trichomes, we used a hole-punch to obtain a 1.8 mm^2^ leaf disc from the leaf tip and counted trichomes on the adaxial (upper) surface of the disc under a dissection microscope. For the chemical traits, we immediately flash froze the remaining portion of the leaf in liquid nitrogen, finely ground it and stored it at −80°C until chemical analysis. All standards required for the chemical measurements were purchased from Sigma-Aldrich (St. Louis, Missouri, USA). Concentration of CHA was determined by ultra-high-performance liquid chromatography (U-HPLC) following the procedure described by Wan and colleagues (2019) [46]. Concentration of kaempferol and rutin were determined by U-HPLC following the procedure described by Wang and colleagues (2012) [64]. Concentration of condensed tannins was determined according to the vanillin–HCL method [65]. Concentration of lignin was determined using the acetyl bromide (AcBr) method [66,67] with modifications as suggested by Yin and colleagues (2023) [47]. Detailed procedures of these chemical analyses are provided in Text B in S1 File.

To measure resistance to herbivory, we used the newly hatched larvae of *S. litura* and adults of *M. hieroglyphica* and *A. sinensis* to conduct bioassays (Table C in S1 File). For *A. sinensis*, we only used males because of the large difference in body size between males and females. Each leaf was first put on a moist filter paper in a petri dish (9 cm in diameter). Then, we released one herbivore individual in each petri dish. Petri dishes were closed and incubated in the growth chamber in the conditions mentioned above. For *S. litura*, we weighed the larva after 5 days; six larvae died during the assay and were, therefore, excluded from subsequent analyses. For the other two herbivores, we took photographs of each leaf using a digital camera after 1 day and estimated the remaining leaf area using Photoshop CS6 software (Adobe Systems, San Jose, CA, USA). Larval weight gain (mg) and leaf area consumed (cm^2^) were used as the measure of plant defense efficacy.

### Experiment 2: costs of defense traits in terms of plant growth

To estimate costs of six selected traits in common ragweed, we assessed the relationships of each trait with plant biomass for plants grown in an unheated greenhouse at Wuhan Botanical Garden (30.54°N, 114.42°E) from April to June 2022 (S1B Fig). The greenhouse was enclosed by a large nylon cage to exclude herbivores, under ambient natural environmental conditions (on average, 13.5 h light/10.5 h dark with 27/18 °C temperature, and relative humidity 50%–100%). We selected two seedlings from each maternal line in each of the above 12 populations (12 populations × 50 maternal lines × 2 replicates = 1,200 plants; Table A in S1 file). We individually transplanted them into 9 L pots (25 cm in height × 22 cm in diameter) filled with a homogenized 50:50 mixture of seedling substrate and sand and supplemented with 10 g slow-release fertilizer (N: P: K = 14: 13: 13; Osmocote, Summerville, SC, USA). After 7 weeks of growth, we collected one fully expanded leaf (the fifth position from the tip) from each plant to measure defense traits. The levels of defense traits were assessed using the same methods as those described in Experiment 1. Then, we harvested the rest of each plant (including above- and belowground parts), dried them at 60 °C for 4 days, and weighed them. Plant fitness is commonly measured in terms of plant growth (*e.g.*, biomass) and/or reproduction (*e.g.*, seed production) [3]. Due to the highly allergenic pollen and rapid dispersal potential of common ragweed, we deliberately harvested plants destructively before flowering. This precluded any direct measurement of reproductive traits and necessitated the use of biomass alone as our proxy for fitness.

### Experiment 3: induction sequence of defense traits with increasing damage

To examine the impacts of damage intensity on the induction threshold of six defense traits, we conducted a growth chamber experiment under the conditions described above from June to August 2023, using plants grown from seeds collected from the HP3 plant population (S1C Fig; Table A in S1 File). Experimental seedlings were randomly selected from 50 maternal lines and planted under the same conditions (pot size and growth substrate) as in Experiment 1. After 10 days of growth, we selected similar size plants with four pairs of fully expanded leaves and exposed each of them to one of 12 generalist chewing insect herbivores (four Coleoptera, four Lepidoptera, and four Orthoptera; Table C in S1 File). For each herbivore, we first enclosed plants individually in nylon mesh cages (40 cm in height, 14 cm in diameter, and 0.125 mm mesh sieve). Then, we randomly assigned them to one of seven herbivore densities (Table C in S1 File) to cause a range of damage from 0% to 100% of leaf area removed which was consistent with leaf damage levels observed in the field [68]. The density of each kind of released herbivore was determined by their consumption capacity. Each density treatment was replicated 18 times (12 herbivore species × 7 densities × 18 replicates = 1,512 plants). Herbivores were allowed to feed for 2 days. Afterwards, we removed herbivores and assessed the total leaf area damaged for each plant (see details in Text C in S1 File).

We used newly grown, fully expanded leaves to measure defense traits because trichomes could not be measured in a constant leaf location for some damaged leaves. As the fifth pair of emerged leaves from the bottom were damaged in some plants during herbivory treatments, we, therefore, selected the sixth pair of leaves from the bottom after about 10 days of growth. For each plant, trichome density was determined as the average of measurements on the sixth pair of leaves (# per 1.8 mm^2^). The levels of five chemical traits were assessed using the rest of this pair of leaves. These defense traits were assessed using the same methods as those described in Experiment 1.

### Statistical analyses

#### Experiment 1.

To investigate the effects of six putative defense traits on the performance of *S. litura*, *M. hieroglyphica*, and *A. sinensis*, we conducted three analyses for each of these three herbivore species. First, we performed a separate LMM for each trait across individual plants from 12 common ragweed populations (n = 72 for *M. hieroglyphica* and *A. sinensis*; n = 66 for *S. litura* after excluding six larval deaths), with herbivore performance (*i.e.*, larval growth or leaf consumption) as the response variable and levels of the trait as the fixed factor along with population as a random factor. Second, because different traits could covary, potentially influencing their individual effects, we used Pearson correlation analyses to examine the relationships among these traits across 72 individual plants. Third, to assess the individual effects of each trait while controlling for the potential influences of other traits, we performed an LMM with herbivore performance as the response variable and all six traits as fixed factors along with population as a random factor. All traits were standardized using Z-scores (mean = 0, SD = 1) before model fitting. We used the variance inflation factor (VIF) to check for multi-collinearity among predictors and found no evidence of severe (VIF > 10) or even moderate (VIF > 5) multi-collinearity (VIF for each predictor < 1.4, Table F in S1 File). In all regression analyses, the response variables (*i.e.*, larval growth or leaf consumption) were log_*e*_-transformed where necessary to improve the fit of model residuals.

#### Experiment 2.

To investigate whether the production of trichomes, tannins, and lignin incurred higher growth costs than CHA, kaempferol, and rutin, we conducted four analyses. First, we performed a separate LMM for each trait across all plant individuals (n = 1,200) from 12 populations, with plant biomass as the response variable and levels of the trait as the fixed factor along with population as a random factor. Second, we performed Pearson correlation analyses to examine covariation among different traits. Third, to assess the individual effect of each trait while controlling for the potential influences of other traits, we conducted an LMM with plant biomass as the response variable and all six traits as fixed factors along with population as a random factor. We standardized each trait using Z-scores (mean = 0, SD = 1) to place them on a comparable scale. Finally, to evaluate the relative impacts of the hypothesized more costly traits (trichomes, tannins, and lignin) and less costly ones (CHA, kaempferol, and rutin) within the LMM with multiple fixed factors, we conducted pairwise comparisons of regression coefficients between each trait in the former group and each trait in the latter group. We inspected for multicollinearity among predictors with the VIF and found very low collinearity (VIF for each predictor < 1.1; Table I in S1 File). For all regression analyses, adding maternal line (nested within population) as a random effect did not improve the model fit; thus, this factor was removed.

#### Experiment 3.

To examine the induction sequence of traits with different costs in response to increasing amounts of damage caused by each herbivore, we conducted a separate regression analysis for each trait with the percentage of leaf area damaged as the explanatory variable and trait levels as the response variable. The treatments of different herbivores were analyzed separately. Concentrations of chemical traits (continuous data) were analyzed using Gaussian models. Trichome densities, initially expressed as the average of two measurements (and, thus, potentially non‑integer), were multiplied by two to ensure all values were integer counts. These counts were analyzed using Poisson models with a log link function. Because high levels of overdispersion were detected for these Poisson models, we refitted the data with quasi-Poisson models [69]. Because defense traits may be induced above a certain threshold of damage, we first fit two models for each trait, one with the percentage of leaf area damaged as a continuous linear predictor and the other with damage as a segmented predictor with a single breakpoint under a null-left-slope constraint [70]. The model with the best fit was identified based on a combination of Davies test [71] and Akaike Information Criterion (AIC, for Gaussian models) or quasi-Akaike Information Criterion (QAIC, for quasi-Poisson models). The segmented model was preferred only if both of the following conditions were met: 1) a significant result from the Davies test (*p* < 0.05) on the linear model and 2) a more than 2-unit lower AIC or QAIC for the segmented model compared to the linear model. If both conditions were not simultaneously met, the linear model was selected.

For the regression analyses conducted for Experiments 1–3, significant effects of the explanatory variables were assessed using *p*-values or confidence intervals (CIs). To control for false discovery rate (FDR) due to multiple comparisons, *p*-values were adjusted using the Benjamini–Hochberg (BH) procedure [72], and CIs were adjusted based on the number of tests and significant results, with an FDR threshold of 0.05 [73]. For the correlation analyses conducted for Experiments 1 and 2, the significance of correlation coefficients was assessed using BH-adjusted *p*-values. All analyses were performed in R, version 4.4.1 [74]. Further details on the analyses are provided in Text D in S1 File.

## Supporting information

S1 File Supplementary results and methods, including Texts A–D and Tables A–M. Text A. Methods of field survey of species composition of insect herbivores. Text B. Methods for chemical analyses. Text C. Method for estimating the total leaf area damaged per plant in Experiment 3. Text D. Further details of statistical analyses. Table A. Locations of *Ambrosia artemisiifolia* populations used in this study. Table B. Field survey of herbivores on *A. artemisiifolia* in July 2019. Data are from four plant populations (30 individuals per population) around Wuhan, China. Detailed survey methods are provided in Text A. For each herbivore, occurrence (# of plants out of 120 surveyed) and the mean abundance per plant where the herbivore occurred are presented. Identification of herbivore species was by morphology, with some further confirmed by DNA-barcoding of cytochrome c oxidase subunit I (CO1). For each species identified with the aid of DNA-barcoding, the highest similarity measure (percent identity) of the specimen’s CO1 sequence compared to sequences in the GenBank database, along with the corresponding GenBank accession number, is provided. Diet breath of herbivores was determined by published references (G = generalist herbivores that can feed on more than one plant family; S = specialist herbivores on Asteraceae; X = specialist on Brassicaceae—accidental occurrence). The data underlying this table can be found in https://doi.org/10.6084/m9.figshare.29364695. Table C. Twelve herbivore species used in this study. For insect bioassays in Experiment 1, the developmental stages of three insects are reported. For herbivory induction treatments in Experiment 3, the developmental stages and the number of individuals used in seven different herbivory treatments (H_1 to H_7) are reported. The acquisition methods of all insects are also provided: CV = commercial vendors; FC = field collection around the study site, Wuhan, in central China. Table D. Results of separate linear mixed models to predict the effect of six plant traits on herbivore performance. The six traits studied, chlorogenic acid (CHA), kaempferol, rutin, trichomes, condensed tannins (tannins), and lignin, were measured in *A. artemisiifolia*. Three herbivore species, *Spodoptera litura*, *Monolepta hieroglyphica*, and *Atractomorpha sinensis*, were used in feeding bioassays. Larval weight gain was measured for *S. litura* after 5 days of feeding, and leaf areas consumed were measured for *M. hieroglyphica* and *A. sinensis* after 1 day of feeding. For each herbivore species, each plant trait was analyzed separately using a linear mixed model (LMM) across individual plants from 12 *A. artemisiifolia* populations (n = 72 for *M. hieroglyphica* and *A. sinensis*; n = 66 for *S. litura* after excluding six larval deaths), with herbivore performance as the response variable and trait levels as the fixed factor along with population as a random factor. Larval weight gain of *S. litura* and leaf area consumed by *M. hieroglyphica* were log_*e*_-transformed. The coefficient estimate for the fixed factor is presented. The statistical significance was estimated by *t* test *p-*values, adjusted using the Benjamini–Hochberg procedure across six separate LMMs for each herbivore. Effects that were significant (adjusted *p* < 0.05) are shown in bold. *R*^2^ (M) and *R*^2^ (C) represent marginal*-* and conditional*-R*^2^, respectively. Table E. Results of pairwise Pearson correlation analyses among six plant traits studied in Experiment 1. The six traits studied, CHA, kaempferol, rutin, trichomes, condensed tannins (tannins), and lignin, were measured in *A. artemisiifolia*. Three sets of plants (n = 72 per set) were each used to evaluate the effects of these traits against one of three herbivore species, *S. litura*, *Monolepta hieroglyphica* and *Atractomorpha sinensis*. For each plant set, all possible pairwise combinations of the six traits (15 pairs in total) were analyzed using Pearson correlation. The correlation coefficient for each trait pair is reported. The statistical significance was estimated by *t* test *p-*values, adjusted using the Benjamini–Hochberg procedure across the 15 pairwise correlations within each plant set. Table F. Results of linear mixed models with multiple fixed factors to predict the effect of six plant traits on herbivore performance. The six traits studied, CHA, kaempferol, rutin, trichomes, condensed tannins (tannins), and lignin, were measured in *A. artemisiifolia*. Three herbivore species, *S. litura*, *M. hieroglyphica*, and *A. sinensis*, were used in feeding bioassays. Larval weight gain was measured for *S. litura* after 5 days of feeding, and leaf areas consumed were measured for *M. hieroglyphica* and *A. sinensis* after 1 day of feeding. Each herbivore was analyzed separately using an LMM across individual plants from 12 *A. artemisiifolia* populations (n = 72 for *M. hieroglyphica* and *A. sinensis*; n = 66 for *S. litura* after excluding six larval deaths), with herbivore performance as the response variable and all six traits as fixed factors along with population as a random factor. Larval weight gain of *S. litura* and leaf area consumed by *M. hieroglyphica* were log_*e*_-transformed. All traits were standardized using Z-scores (mean = 0, SD = 1) before model fitting. The variance inflation factor (VIF) and coefficient estimate for each fixed factor are presented. The statistical significance was estimated by *t* test *p-*values, adjusted using the Benjamini–Hochberg procedure across all tests within each model. Effects that were significant (adjusted *p* < 0.05) are shown in bold. *R*^2^ (M) and *R*^2^ (C) represent marginal*-* and conditional*-R*^2^, respectively. Table G. Results of separate linear mixed models to predict the effect of six plant traits on plant biomass. The six traits studied, CHA, kaempferol, rutin, trichomes, condensed tannins (tannins), and lignin, were measured in *A. artemisiifolia*. Each trait was analyzed separately using an LMM across 1,200 individual plants from all 12 *A. artemisiifolia* populations, with plant biomass as the response variable and trait levels as the fixed factor along with population as a random factor. The coefficient estimate for the fixed factor is presented. The statistical significance was estimated by *t* test *p*-values, adjusted using the Benjamini–Hochberg procedure across six separate LMMs. Effects that were significant (adjusted *p* < 0.05) are shown in bold. *R*^2^ (M) and *R*^2^ (C) represent marginal*-* and conditional*-R*^2^, respectively. Table H. Results of pairwise Pearson correlation analyses among six plant traits studied in Experiment 2. The six traits studied, CHA, kaempferol, rutin, trichomes, condensed tannins (tannins), and lignin, were measured in *A. artemisiifolia* plants (n = 1,200) used to evaluate the growth costs of traits. All possible pairwise combinations of the six traits (15 pairs in total) were analyzed using Pearson correlation. The correlation coefficient for each trait pair is reported. The statistical significance was estimated by *t* test *p-*values, adjusted using the Benjamini–Hochberg procedure across the 15 pairwise correlations. Effects that were significant (adjusted *p* < 0.05) are shown in bold. Table I. Results of a linear mixed model with multiple fixed factors to predict the effect of six plant traits on plant biomass. The six traits studied, CHA, kaempferol, rutin, trichomes, condensed tannins (tannins), and lignin, were measured in *A. artemisiifolia*. The analysis was conducted using an LMM across 1,200 individual plants from all 12 *A. artemisiifolia* populations, with plant biomass as the response variable and all six traits as fixed factors along with population as a random factor. All trait levels data were Z-score-transformed. The VIF and coefficient estimate for each fixed factor are presented. The statistical significance was estimated by *t* test *p*-values, adjusted using the Benjamini–Hochberg procedure across all tests. Effects that were significant (adjusted *p* < 0.05) are shown in bold. *R*^2^ (M) and *R*^2^ (C) represent marginal*-* and conditional*-R*^2^, respectively. Table J. Results of pairwise comparisons in regression slopes for traits analyzed in the linear mixed model provided in Table I. Regression slopes were individually compared between each of CHA, kaempferol, and rutin and each of trichomes, condensed tannins (tannins), and lignin using linear hypothesis tests. Specifically, for each pair of traits, we tested the null hypothesis that the slopes of two traits were equal by comparing a restricted model, in which the slopes of the two traits were constrained to be equal, with the original LMM. The difference in model fit was evaluated using a chi-squared test, and the significance of the slope difference was determined by the *p*-value from the chi-squared test. The *p*-values were corrected across all pairwise comparisons using Benjamini–Hochberg method. Effects that were significant (adjusted *p* < 0.05) are shown in bold. The differences in slopes between each pair of traits are presented. DF represents the difference in degrees of freedom between the constrained and original models. Table K. Population‑specific effects of six plant traits on plant biomass, extracted from a single multiple linear model. The six traits studied, CHA, kaempferol, rutin, trichomes, condensed tannins (tannins), and lignin, were measured in *A. artemisiifolia*. The analysis was conducted using a multiple linear model across 1,200 individual plants from all 12 *A. artemisiifolia* populations. Plant biomass was used as the response variable, while all six traits, population and all trait × population interactions were used as explanatory variables. Adding maternal line (nested within population) as a random effect did not improve the model fit, thus this factor was removed. All trait levels data were Z-score-transformed. Coefficient estimates for each trait within each population are presented. The statistical significance was estimated by *t* test *p*-values, adjusted using the Benjamini–Hochberg procedure across all tests. Effects that were significant (adjusted *p* < 0.05) are shown in bold. Table L. Results of regression analyses for herbivory intensity on the levels of six plant traits. Twelve insect species were used to impose herbivory treatments on *A. artemisiifolia*. For each herbivore treatment, the six plant traits studied, CHA, kaempferol, rutin, trichomes, condensed tannins (tannins), and lignin, were analyzed separately. Trichome densities were analyzed using quasi-Poisson models and other traits were analyzed using Gaussian models. Herbivory intensity (the percentage of leaf area damaged) was included as a linear or segmented term with a single breakpoint under a null-left-slope constraint. The model with the best fit was identified based on a combination of Davies test and Akaike Information Criterion (AIC) or quasi-AIC (QAIC). Coefficient estimates [Linear models: slopes for the whole damage range; Segmented models: break points with 95% confidence intervals (CIs) and slopes after break points] are presented. The goodness of fit for Gaussian models are reported as *R*^2^ and that for quasi-Poisson models are reported as explained deviances: [1 – (residual deviance/null deviance)]. The statistical significance of the slopes was estimated by CIs from *t* tests. Across all analyses for each herbivore, CIs were corrected for multiple comparisons based on the number of tests and the number of significant results at a false discovery rate threshold of 0.05. Significant effects (*i.e.*, adjusted 95% CIs not crossing zero) are shown in bold. SE = standard errors for the regression slopes. Table M. Regression slope comparisons in linear mixed models with multiple fixed factors predicting effects of six plant traits on herbivore performance. The six traits studied, CHA, kaempferol, rutin, trichomes, condensed tannins (tannins), and lignin, were measured in *A. artemisiifolia*. Three herbivore species, *S. litura*, *M. hieroglyphica*, and *A. sinensis* were used in feeding bioassays. The data and analytical procedures were identical to those used in Table F. Traits were classified into two groups based on their induction patterns in response to increasing herbivore damage in Experiment 3: group 1 exhibited a linear response, while group 2 showed a segmented response. Traits not induced in Experiment 3 were excluded from subsequent analyses. Within each model, the average regression slopes of the two groups were compared using linear hypothesis tests. Specifically, we tested the null hypothesis that the slopes of the two groups were equal by comparing a restricted model, in which the slopes of the two groups were constrained to be equal, with the original model. The difference in model fit was evaluated using a chi-squared test, and the significance of the slope difference was determined by the *p*-value from the chi-squared test. The differences in average slopes between the two groups of traits are presented. DF represents the differences in degrees of freedom between the constrained and original models.
(DOCX)

S1 Fig Schematic depiction of three experiments in this study. Experiment 1 **(A)** assessed the defense efficacy of six putative defense traits. We collected the sixth leaf pair (from the bottom) of 30-day-old plants: one leaf was used to quantify six putative defense traits, and the other to assess resistance to herbivory. This procedure was repeated three times, each with a different insect herbivore species. Experiment 2 **(B)** assessed the growth costs of six defense traits. We collected the fifth leaf (from the tip) of 59-day-old plants to measure six defense traits, then harvested, dried and weighed the rest (including above- and belowground parts). Experiment 3 **(C)** assessed induction sequence of defense traits with increasing herbivory. We exposed 20-day-old plants to each of 12 generalist herbivore species at seven increasing density levels (Density 1–7). After 2 days of feeding, insects were removed, and damage severity was recorded. After 10 days of growth, we collected the sixth leaf pair (from the bottom) for defense‐trait measurements. Information on plant materials and herbivore species is provided in Tables A and B in S1 File, respectively.
(TIF)

S2 Fig Lower-cost plant traits are induced at lower levels of damage caused by *Spodoptera litura.* Relationships between levels of each of six traits, chlorogenic acid (CHA), kaempferol, rutin, trichomes, condensed tannins (tannins), and lignin, in *Ambrosia artemisiifolia* and the percentage of leaf area damaged by *S. litura*. Each trait was analyzed separately. The reaction norm for each trait was chosen between a linear or segmented model based on a combination of the Davies test and AIC/QAIC. Data points represent individual replicates (n = 18 per density treatment, seven density treatments). The different line colors represent different traits. Solid lines indicate significant effects (adjusted 95% confidence intervals not crossing zero) of herbivore damage on levels of traits. Predicted trichome densities are presented on the original scale (*i.e.*, # per 1.8 mm^2^). Statistical results are in Table L in S1 File. FM, leaf fresh biomass. CW, cell wall. The data underlying this figure can be found in https://doi.org/10.6084/m9.figshare.29364695.
(TIF)

S3 Fig Lower-cost plant traits are induced at lower levels of damage caused by *Spodoptera exigua.* Relationships between levels of each of six traits, chlorogenic acid (CHA), kaempferol, rutin, trichomes, condensed tannins (tannins), and lignin, in *Ambrosia artemisiifolia* and the percentage of leaf area damaged by *S*. *exigua*. Each trait was analyzed separately. The reaction norm for each trait was chosen between a linear or segmented model based on a combination of the Davies test and AIC/QAIC. Data points represent individual replicates (n = 18 per density treatment, seven density treatments). The different line colors represent different traits. Solid lines indicate significant effects (adjusted 95% confidence intervals not crossing zero) of herbivore damage on levels of traits while dotted lines represent nonsignificant effects (adjusted 95% confidence intervals crossing zero). Predicted trichome densities are presented on the original scale (*i.e.*, # per 1.8 mm^2^). Statistical results are in Table L in S1 File. FM, leaf fresh biomass. CW, cell wall. The data underlying this figure can be found in https://doi.org/10.6084/m9.figshare.29364695.
(TIF)

S4 Fig Lower-cost plant traits are induced at lower levels of damage caused by *Helicoverpa armigera.* Relationships between levels of each of six traits, chlorogenic acid (CHA), kaempferol, rutin, trichomes, condensed tannins (tannins), and lignin, in *Ambrosia artemisiifolia* and the percentage of leaf area damaged by *H. armigera*. Each trait was analyzed separately. The reaction norm for each trait was chosen between a linear or segmented model based on a combination of the Davies test and AIC/QAIC. Data points represent individual replicates (n = 18 per density treatment, seven density treatments). The different line colors represent different traits. Solid lines indicate significant effects (adjusted 95% confidence intervals not crossing zero) of herbivore damage on levels of traits while dotted lines represent nonsignificant effects (adjusted 95% confidence intervals crossing zero). Predicted trichome densities are presented on the original scale (*i.e.*, # per 1.8 mm^2^). Statistical results are in Table L in S1 File. FM, leaf fresh biomass. CW, cell wall. The data underlying this figure can be found in https://doi.org/10.6084/m9.figshare.29364695.
(TIF)

S5 Fig Lower-cost plant traits are induced at lower levels of damage caused by *Spodoptera frugiperda.* Relationships between levels of each of six traits, chlorogenic acid (CHA), kaempferol, rutin, trichomes, condensed tannins (tannins), and lignin, in *Ambrosia artemisiifolia* and the percentage of leaf area damaged by *S. frugiperda*. Each trait was analyzed separately. The reaction norm for each trait was chosen between a linear or segmented model based on a combination of the Davies test and AIC/QAIC. Data points represent individual replicates (n = 18 per density treatment, seven density treatments). The different line colors represent different traits. Solid lines indicate significant effects (adjusted 95% confidence intervals not crossing zero) of herbivore damage on levels of traits while dotted lines represent nonsignificant effects (adjusted 95% confidence intervals crossing zero). Predicted trichome densities are presented on the original scale (*i.e.*, # per 1.8 mm^2^). Statistical results are in Table L in S1 File. FM, leaf fresh biomass. CW, cell wall. The data underlying this figure can be found in https://doi.org/10.6084/m9.figshare.29364695.
(TIF)

S6 Fig Lower-cost plant traits are induced at lower levels of damage caused by *Monolepta hieroglyphica.* Relationships between levels of each of six traits, chlorogenic acid (CHA), kaempferol, rutin, trichomes, condensed tannins (tannins), and lignin, in *Ambrosia artemisiifolia* and the percentage of leaf area damaged by *M. hieroglyphica*. Each trait was analyzed separately. The reaction norm for each trait was chosen between a linear or segmented model based on a combination of the Davies test and AIC/QAIC. Data points represent individual replicates (n = 18 per density treatment, seven density treatments). The different line colors represent different traits. Solid lines indicate significant effects (adjusted 95% confidence intervals not crossing zero) of herbivore damage on levels of traits while dotted lines represent nonsignificant effects (adjusted 95% confidence intervals crossing zero). Predicted trichome densities are presented on the original scale (*i.e.*, # per 1.8 mm^2^). Statistical results are in Table L in S1 File. FM, leaf fresh biomass. CW, cell wall. The data underlying this figure can be found in https://doi.org/10.6084/m9.figshare.29364695.
(TIF)

S7 Fig Lower-cost plant traits are induced at lower levels of damage caused by *Luperomorpha xanthodera.* Relationships between levels of each of six traits, chlorogenic acid (CHA), kaempferol, rutin, trichomes, condensed tannins (tannins), and lignin, in *Ambrosia artemisiifolia* and the percentage of leaf area damaged by *L. xanthodera*. Each trait was analyzed separately. The reaction norm for each trait was chosen between a linear or segmented model based on a combination of the Davies test and AIC/QAIC. Data points represent individual replicates (n = 18 per density treatment, seven density treatments). The different line colors represent different traits. Solid lines indicate significant effects (adjusted 95% confidence intervals not crossing zero) of herbivore damage on levels of traits while dotted lines represent nonsignificant effects (adjusted 95% confidence intervals crossing zero). Predicted trichome densities are presented on the original scale (*i.e.*, # per 1.8 mm^2^). Statistical results are in Table L in S1 File. FM, leaf fresh biomass. CW, cell wall. The data underlying this figure can be found in https://doi.org/10.6084/m9.figshare.29364695.
(TIF)

S8 Fig Lower-cost plant traits are induced at lower levels of damage caused by *Nonarthra cyaneum.* Relationships between levels of each of six traits, chlorogenic acid (CHA), kaempferol, rutin, trichomes, condensed tannins (tannins), and lignin, in *Ambrosia artemisiifolia* and the percentage of leaf area damaged by *N. cyaneum*. Each trait was analyzed separately. The reaction norm for each trait was chosen between a linear or segmented model based on a combination of the Davies test and AIC/QAIC. Data points represent individual replicates (n = 18 per density treatment, seven density treatments). The different line colors represent different traits. Solid lines indicate significant effects (adjusted 95% confidence intervals not crossing zero) of herbivore damage on levels of traits while dotted lines represent nonsignificant effects (adjusted 95% confidence intervals crossing zero). Predicted trichome densities are presented on the original scale (*i.e.*, # per 1.8 mm^2^). Statistical results are in Table L in S1 File. FM, leaf fresh biomass. CW, cell wall. The data underlying this figure can be found in https://doi.org/10.6084/m9.figshare.29364695.
(TIF)

S9 Fig Lower-cost plant traits are induced at lower levels of damage caused by *Piazomias fausti.* Relationships between levels of each of six traits, chlorogenic acid (CHA), kaempferol, rutin, trichomes, condensed tannins (tannins), and lignin, in *Ambrosia artemisiifolia* and the percentage of leaf area damaged by *P. fausti*. Each trait was analyzed separately. The reaction norm for each trait was chosen between a linear or segmented model based on a combination of the Davies test and AIC/QAIC. Data points represent individual replicates (n = 18 per density treatment, seven density treatments). The different line colors represent different traits. Solid lines indicate significant effects (adjusted 95% confidence intervals not crossing zero) of herbivore damage on levels of traits while dotted lines represent nonsignificant effects (adjusted 95% confidence intervals crossing zero). Predicted trichome densities are presented on the original scale (*i.e.*, # per 1.8 mm^2^). Statistical results are in Table L in S1 File. FM, leaf fresh biomass. CW, cell wall. The data underlying this figure can be found in https://doi.org/10.6084/m9.figshare.29364695.
(TIF)

S10 Fig Lower-cost plant traits are induced at lower levels of damage caused by *Atractomorpha sinensis.* Relationships between levels of each of six traits, chlorogenic acid (CHA), kaempferol, rutin, trichomes, condensed tannins (tannins), and lignin, in *Ambrosia artemisiifolia* and the percentage of leaf area damaged by *A. sinensis*. Each trait was analyzed separately. The reaction norm for each trait was chosen between a linear or segmented model based on a combination of the Davies test and AIC/QAIC. Data points represent individual replicates (n = 18 per density treatment, seven density treatments). The different line colors represent different traits. Solid lines indicate significant effects (adjusted 95% confidence intervals not crossing zero) of herbivore damage on levels of traits while dotted lines represent nonsignificant effects (adjusted 95% confidence intervals crossing zero). Predicted trichome densities are presented on the original scale (*i.e.*, # per 1.8 mm^2^). Statistical results are in Table L in S1 File. FM, leaf fresh biomass. CW, cell wall. The data underlying this figure can be found in https://doi.org/10.6084/m9.figshare.29364695.
(TIF)

S11 Fig Lower-cost plant traits are induced at lower levels of damage caused by *Oecanthus rufescens.* Relationships between levels of each of six traits, chlorogenic acid (CHA), kaempferol, rutin, trichomes, condensed tannins (tannins), and lignin, in *Ambrosia artemisiifolia* and the percentage of leaf area damaged by *O. rufescens.* Each trait was analyzed separately. The reaction norm for each trait was chosen between a linear or segmented model based on a combination of the Davies test and AIC/QAIC. Data points represent individual replicates (n = 18 per density treatment, seven density treatments). The different line colors represent different traits. Solid lines indicate significant effects (adjusted 95% confidence intervals not crossing zero) of herbivore damage on levels of traits while dotted lines represent nonsignificant effects (adjusted 95% confidence intervals crossing zero). Predicted trichome densities are presented on the original scale (*i.e.*, # per 1.8 mm^2^). Statistical results are in Table L in S1 File. FM, leaf fresh biomass. CW, cell wall. The data underlying this figure can be found in https://doi.org/10.6084/m9.figshare.29364695.
(TIF)

S12 Fig Lower-cost plant traits are induced at lower levels of damage caused by *Xenocatantops brachycerus.* Relationships between levels of each of six traits, chlorogenic acid (CHA), kaempferol, rutin, trichomes, condensed tannins (tannins), and lignin, in *Ambrosia artemisiifolia* and the percentage of leaf area damaged by *X. brachycerus*. Each trait was analyzed separately. The reaction norm for each trait was chosen between a linear or segmented model based on a combination of the Davies test and AIC/QAIC. Data points represent individual replicates (n = 18 per density treatment, seven density treatments). The different line colors represent different traits. Solid lines indicate significant effects (adjusted 95% confidence intervals not crossing zero) of herbivore damage on levels of traits while dotted lines represent nonsignificant effects (adjusted 95% confidence intervals crossing zero). Predicted trichome densities are presented on the original scale (*i.e.*, # per 1.8 mm^2^). Statistical results are in Table L in S1 File. FM, leaf fresh biomass. CW, cell wall. The data underlying this figure can be found in https://doi.org/10.6084/m9.figshare.29364695.
(TIF)

S13 Fig Lower-cost plant traits are induced at lower levels of damage caused by *Phaneroptera falcata.* Relationships between levels of each of six traits, chlorogenic acid (CHA), kaempferol, rutin, trichomes, condensed tannins (tannins), and lignin, in *Ambrosia artemisiifolia* and the percentage of leaf area damaged by *P. falcata*. Each trait was analyzed separately. The reaction norm for each trait was chosen between a linear or segmented model based on a combination of the Davies test and AIC/QAIC. Data points represent individual replicates (n = 18 per density treatment, seven density treatments). The different line colors represent different traits. Solid lines indicate significant effects (adjusted 95% confidence intervals not crossing zero) of herbivore damage on levels of traits while dotted lines represent nonsignificant effects (adjusted 95% confidence intervals crossing zero). Predicted trichome densities are presented on the original scale (*i.e.*, # per 1.8 mm^2^). Statistical results are in Table L in S1 File. FM, leaf fresh biomass. CW, cell wall. The data underlying this figure can be found in https://doi.org/10.6084/m9.figshare.29364695.
(TIF)

## Abbreviations

AIC: Akaike Information Criterion
BH: Benjamini–Hochberg
CHA: chlorogenic acid
CI: confidence interval
DM: plant dry biomass
FDR: false discovery rate
FM: leaf fresh biomass
CW: cell wall
LMM: linear mixed model
QAIC: quasi-Akaike Information Criterion
U-HPLC: ultra-high-performance liquid chromatography
VIF: variance inflation factor.
