# Supplementary material for: Plants respond to herbivory through sequential induction of cheaper defenses before more costly ones
Source: PLoS Biol. 2025 Aug 14;23(8):e3003280. doi: 10.1371/journal.pbio.3003280 (PMC12352644; doi:10.1371/journal.pbio.3003280)
Supplement: S1 File — Text A. Methods of field survey of species composition of insect herbivores. Text B. Methods for chemical analyses. Text C. Method for estimating the total leaf area damaged per plant in Experiment 3. Text D. Further details of statistical analyses. Table A. Locations of Ambrosia artemisiifolia populations used in this study. Table B. Field survey of herbivores on A. artemisiifolia in July 2019. Data are from four plant populations (30 individuals per population) around Wuhan, China. Detailed survey methods are provided in Text A. For each herbivore, occurrence (# of plants out of 120 surveyed) and the mean abundance per plant where the herbivore occurred are presented. Identification of herbivore species was by morphology, with some further confirmed by DNA-barcoding of cytochrome c oxidase subunit I (CO1). For each species identified with the aid of DNA-barcoding, the highest similarity measure (percent identity) of the specimen’s CO1 sequence compared to sequences in the GenBank database, along with the corresponding GenBank accession number, is provided. Diet breath of herbivores was determined by published references (G = generalist herbivores that can feed on more than one plant family; S = specialist herbivores on Asteraceae; X = specialist on Brassicaceae—accidental occurrence). The data underlying this table can be found in https://doi.org/10.6084/m9.figshare.29364695. Table C. Twelve herbivore species used in this study. For insect bioassays in Experiment 1, the developmental stages of three insects are reported. For herbivory induction treatments in Experiment 3, the developmental stages and the number of individuals used in seven different herbivory treatments (H_1 to H_7) are reported. The acquisition methods of all insects are also provided: CV = commercial vendors; FC = field collection around the study site, Wuhan, in central China. Table D. Results of separate linear mixed models to predict the effect of six plant traits on herbivore performance. Th [file pbio.3003280.s001.docx]

**S1 File - Supporting Information**

**Plants respond to herbivory through sequential induction of cheaper defenses before more costly ones**

Jinlong Wan^1,2^, Jiahui Yi^1,2,3^, Xiao Sun^4^, Evan Siemann^5^, Matthias Erb^6^, Wei Huang^1,2*^

^1^ Wuhan Botanical Garden, Chinese Academy of Sciences, Wuhan, China.

^2^ Hubei Key Laboratory of Wetland Evolution & Ecological Restoration, Wuhan Botanical Garden, Chinese Academy of Sciences, Wuhan, China.

^3^ University of Chinese Academy of Sciences, Beijing, China.

^4^ School of Life Sciences, Henan University, Kaifeng, China.

^5^ Department of Biosciences, Rice University, Houston, Texas, USA.

^6^ Institute of Plant Sciences, University of Bern, Bern, Switzerland.

* huangwei0519@wbgcas.cn (WH)

**This file includes:**

Text A-D.

Tables A-M.

References.

**Text A. Methods of field survey of species composition of insect herbivores.**

To determine the species composition of insect herbivores on *Ambrosia artemisiifolia* around Wuhan, China, we conducted a field survey of four plant populations (HP3: 114.39°E, 30.92°N; XZ1: 114.74°E, 30.84°N; JX1:114.53°E, 30.50°N; WN: 114.02°E, 30.27°N) in July 2019. In each population, we randomly selected 30 plants (80-120 cm in height, separated by at least 10 m) along farmland roadsides. We used a mesh bag (160 cm in height, 57.3 cm in diameter) to collect herbivores on the aboveground portion of each plant. Insects that were not Lepidoptera were transferred into tubes with 80% ethanol. Lepidoptera larvae were taken back alive to the laboratory and reared to adults on cut *A. artemisiifolia* leaves. All specimens were identified to species based on morphology where possible by taxonomic experts (see Acknowledgements). Some specimen identifications were further confirmed by DNA barcoding when morphological identifications to species were uncertain. For DNA extraction, we used 2×Taq PCR Master Mix (Gene Tech, Shanghai, China). We amplified the barcoding region of the mitochondrial Cytochrome Oxidase I (COI) gene with the universal primers LCO 1490 and HCO 2198 [1]. Products were sequenced with the 3730xl DNA Analyzer (Applied Biosystems, Carlsbad, CA, USA). Sequences were analyzed using SeqMan 7.1 (DNAStar, Madison, WI, USA) and Chromas 2.6.5 (Technelysium, South Brisbane, Australia). The resulting sequences were compared with the GenBank database using the Basic Local Alignment Search Tool (BLAST). A species-level taxonomic identity was assigned to a specimen when the sequence of this specimen was >97% similar (percent identity) to a sequence referenced in the database.

**Text B. Methods for chemical analyses.**

Concentration of CHA was determined by ultra-high performance liquid chromatography (U-HPLC) following the procedure described by Wan *et al.* (2019) [2] with a CHA standard. The leaf powder (50 mg) was soaked in a 0.5 ml of a solution of methanol‐0.4% phosphoric acid in water (50:50, v:v) for 24 hr. After sonication, centrifugation, and filtration, 20 μL of extract was injected into an Agilent 1290 Infinity II UHPLC (Agilent Technologies, Waldbronn, Germany) equipped with a Thermo Hypersil GOLD C18 column (250 × 4.6 mm, 5 μm; Thermo Fisher Scientific, Waltham, MA, USA). The mobile phase solution consisted of acetonitrile/0.4% phosphoric acid (A; 10:90, v:v) and 100% acetonitrile (B). The flow rate was 1.0 ml/min, with an A‐B gradient as follows: 0–10 min, 100:0; 10–15 min, 80:20; 15–20 min, 0:100. UV absorbance spectra were monitored at 326 nm. The results were expressed as μg g^-1^.

Concentration of kaempferol and rutin were determined by U-HPLC following the procedure described by Wang *et al.* (2012) [3], with kaempferol and rutin trihydrate used as the respective standards. The extract (20 μL) required for the measurement was taken from the filtrate already prepared during the CHA determination mentioned above. The equipment needed for the measurement was the same as those used for measuring CHA. The mobile phase solution consisted of 0.4% phosphoric acid (A) and 100% methanol (B). The flow rate was 1.0 ml/min, with an A‐B gradient as follows: 0–10 min, 50:50; 10–18.5 min, 35:65. UV absorbance spectra were monitored at 254 nm. The results were expressed as μg g^-1^.

Concentration of condensed tannins was determined according to the vanillin-HCL method [4]. 50 mg of leaf powder was suspended in 1 mL of methanol solution, shaken at room temperature for 1 hr, and then centrifuged at 1000 g for 10 min. 200 μL of the supernatant was mixed with 1.8 mL of vanillin reagent (comprising 4% (w/v) vanillin and 10% (v/v) HCl in methanol) and incubated at 30°C for 20 min in the dark. The absorbance was then measured at 500 nm on an Infinite 200 PRO plate reader (Tecan Austria GmbH, Grödig, Austria). Catechin was used as a standard. The results were expressed as mg g^−1^.

Concentration of lignin was determined using the acetyl bromide (AcBr) method [5,6] with modifications as suggested by Yin *et al.* (2023) [7]. To exclude protein and other UV-absorbing materials, the leaf powder was suspended in 50 mM sodium phosphate buffer (7ml, pH 7.0), and washed with phosphate buffer (two times; pH 7.0; 7 ml), 1% (v/v) Triton X-100 (three times; pH 7.0; 7ml), 1M NaCl (two times; pH 7.0; 7 ml), distilled water (two times; 7ml), ethanol (two times; 70%; 7ml), and acetone (two times; 5ml). The pellet was air-dried for 24 hr and the remaining protein-free cell wall (CW) fraction was used for quantifying lignin content. The CW fraction (1 mg) was digested with 0.5 mL of a 25% acetyl bromide in acetic acid solution and incubated at 70 ℃ for 30 min. The sample was rapidly cooled in an ice bath, and then mixed with 0.9 ml of 2 M NaOH, 0.1 ml of 5 M hydroxylamine-HCl. Acetic acid was added to bring up the solution to 10 ml. To ensure accurate readings on the plate reader (as described in the tannins measurement), the above solution was further diluted five times with acetic acid. After centrifugation (1400 g, 5 min), the absorbance of the mixture was read at 280 nm. Alkali lignin was used as a standard. The results were expressed as mg g^−1^ CW.

**Text C. Method for estimating the total leaf area damaged per plant in Experiment 3.**

For each plant, the initial leaf area was nearly equal between the two leaves within each pair but varied substantially across pairs. An additional assessment of 50 HP3 plants, each with four fully expanded leaf pairs and grown under identical conditions as the experimental plants, showed that the first to fourth leaf pairs (from bottom to top) accounted for 6%, 18%, 42%, and 34% of total leaf area, respectively. The individual percentage values are provided in https://doi.org/10.6084/m9.figshare.29364695. To accurately estimate the damage for each plant, we first visually assessed the damage to each leaf using a 5% interval category (*e.g.*, 0%, 5%, 10%). The estimated damage for each leaf was then multiplied by its respective proportion of the total leaf area. Finally, these weighted values were summed across all leaves to calculate the total damage for the plant.

**Text D. Further details of statistical analyses.**

**Experiment 1**. For the regression analyses, significant effects of the explanatory variables were assessed by *p*-values from *t* tests. To control for false discovery rate (FDR) due to multiple comparisons, we applied the Benjamini–Hochberg (BH) procedure [8] to adjust *p*-values across six separate LMMs and within the LMM with multiple fixed factors for each herbivore. Goodness-of-fit of models were reported as marginal*-* and conditional-*R*^2^ [9]. For the correlation analyses, the significance of correlation coefficients was assessed by *t*-test *p*-values, adjusted using the BH method across all pairwise correlations (15 trait pairs) for each herbivore.

**Experiment 2.** For the regression analyses, significant effects of the explanatory variables were assessed by *t*-test *p*-values, adjusted using the BH procedure across six separate LMMs and within the LMM with multiple fixed factors. Goodness-of-fit of models were reported as marginal*-* and conditional-*R*^2^. Pairwise comparisons of regression slopes in the LMM with multiple fixed factors were performed using linear hypothesis tests. Specifically, for each pair of traits, we tested the null hypothesis that the slopes of two traits were equal by comparing a restricted model, in which the slopes of the two traits were constrained to be equal, with the original LMM model. The difference in model fit was evaluated using a chi-square test, and the resulting *p*-values were BH-corrected across all pairwise comparisons. For the correlation analyses, the significance of correlation coefficients was assessed by *t*-test *p*-values, adjusted using BH method across all 15 trait pairs.

**Experiment 3.** For the regression analyses, significant effects of the explanatory variables were assessed by confidence intervals (CIs) from *t* tests. Across all analyses for each herbivore, we adjusted CIs for multiple comparisons based on the number of tests and the number of significant results at an FDR threshold of 0.05 [10]. Specifically, the adjusted CI was calculated as 1 – q×R/m, where q is the level at which the FDR is controlled (0.05), R is the number of significant tests at this FDR threshold (0.05), and m is the total number of tests performed. For the multiple comparisons in Davies tests, *p*-values were not adjusted, as the computed values provide an upper bound and the tests are inherently conservative [11]. Goodness-of-fit of models were reported as *R*^2^ in Gaussian models and as [1 – (residual deviance/null deviance)] in quasi-Poisson models.

All analyses were performed in R, version 4.4.1 [12]. The package ‘stats’ was used to fit the linear models, perform Pearson correlations, and adjust *p*-values. The package ‘nlme’ was used to conduct the LMMs [13]. The package ‘segmented’ was used to conduct Davies test and fit the segmented regressions [14]. The package ‘MuMIn’ was used to calculate the marginal *R*^2^ and the conditional *R*^2^ [15]. The package ‘bbmle’ was used to calculate QAIC [16]. The package ‘car’ was used to check multi-collinearity and compare regression coefficients of predictors in LMMs with multiple fixed factors [17].

**Table A.** **Locations of *Ambrosia artemisiifolia* populations used in this study.**

| **Populations** | **Locations** | **Longitude (°E)** | **Latitude (°N)** |
| --- | --- | --- | --- |
| HP1 | Huangpi | 114.34 | 31.04 |
| HP2 | Huangpi | 114.57 | 30.93 |
| HP3 | Huangpi | 114.39 | 30.92 |
| XZ1 | Xinzhou | 114.74 | 30.84 |
| XZ2 | Xinzhou | 114.63 | 30.69 |
| DXH | Dongxihu | 114.18 | 30.64 |
| JX1 | Jiangxia | 114.53 | 30.50 |
| JX2 | Jiangxia | 114.55 | 30.39 |
| JX3 | Jiangxia | 114.34 | 30.33 |
| JX4 | Jiangxia | 114.5 | 30.14 |
| CD | Caidian | 113.96 | 30.46 |
| WN | Wennan | 114.02 | 30.27 |

**Table B. Field survey of herbivores on *Ambrosia artemisiifolia* in July 2019.** Data are from four plant populations (30 individuals per population) around Wuhan, China. Detailed survey methods are provided in Text A. For each herbivore, occurrence (# of plants out of 120 surveyed) and the mean abundance per plant where the herbivore occurred are presented. Identification of herbivore species was by morphology, with some further confirmed by DNA-barcoding of cytochrome c oxidase subunit I (CO1). For each species identified with the aid of DNA-barcoding, the highest similarity measure (percent identity) of the specimen’s CO1 sequence compared to sequences in the GenBank database, along with the corresponding GenBank accession number, is provided. Diet breath of herbivores was determined by published references (G = generalist herbivores that can feed on more than one plant family; S = specialist herbivores on Asteraceae; X = specialist on Brassicaceae – accidental occurrence). The data underlying this table can be found in https://doi.org/10.6084/m9.figshare.29364695.

| **Orders** | **Species** | **Occurrence (# of plants)** | **Abundance per plant (no.)** | **Similarity measure of COI (%)** | **GenBank accession numbers** | **Diet breath** | **References about diet breath** |
| --- | --- | --- | --- | --- | --- | --- | --- |
| Lepidoptera | *Condica illecta* | 3 | 1.33 | 100.00 | SUB15078358 Seq7 PV083095 | S | [18] |
| Lepidoptera | *Helicoverpa armigera* | 10 | 1.10 | 100.00 | SUB15078358 Seq8 PV083096 | G | [19] |
| Lepidoptera | *Spodoptera litura* | 10 | 1.20 |  |  | G | [20] |
| Lepidoptera | *Epiblema strenuana* | 15 | 1.20 |  |  | S^1^ | [21] |
| Coleoptera | *Luperomorpha xanthodera* | 3 | 1.33 | 99.34 | SUB15078358 Seq10 PV083098 | G | [22] |
| Coleoptera | *Monolepta hieroglyphica* | 8 | 1.50 |  |  | G | [23] |
| Coleoptera | *Nonarthra cyaneum* | 2 | 1.50 |  |  | G | [24] |
| Coleoptera | *Ophraella communa* | 90 | 44.11 |  |  | S | [25] |
| Coleoptera | *Phyllotreta striolata* | 1 | 1.00 |  |  | X | [26] |
| Coleoptera | *Piazomias fausti* | 19 | 1.26 |  |  | G | [27] |
| Orthoptera | *Acrida cinerea* | 3 | 1.00 | 99.68 | SUB15078358 Seq1 PV083089 | G | [28] |
| Orthoptera | *Aiolopus thalassinus tamulus* | 2 | 1.00 | 100.00 | SUB15078358 Seq3 PV083091 | G | [29] |
| Orthoptera | *Xenocatantops* *brachycerus* | 15 | 1.13 | 99.53 | SUB15078358 Seq15 PV083103 | G | [30] |
| Orthoptera | *Chondracris rosea* | 2 | 1.00 | 99.84 | SUB15078358 Seq6 PV083094 | G | [31] |
| Orthoptera | *Oecanthus rufescens* | 33 | 1.82 | 100.00 | SUB15078358 Seq12 PV083100 | G | [32] |
| Orthoptera | *Atractomorpha sinensis* | 43 | 1.72 | 99.84 | SUB15078358 Seq5 PV083093 | G | [33] |
| Orthoptera | *Phaneroptera falcata* | 9 | 1.67 | 99.67 | SUB15078358 Seq13 PV083101 | G | [34] |
| Hemiptera | *Leptocorisa oratoria* | 12 | 1.17 | 100.00 | SUB15078358 Seq9 PV083097 | G | [35] |
| Hemiptera | *Riptortus pedestris* | 8 | 1.00 | 99.50 | SUB15078358 Seq14 PV083102 | G | [36] |
| Hemiptera | *Aphis gossypii* | 15 |  | 100.00 | SUB15078358 Seq4 PV083092 | G | [37] |
| Hemiptera | *Hemiptera sp.* | 17 | 5.88 |  |  |  |  |
| Hemiptera | *Nysius inconspicuous* | 42 | 1.88 | 100.00 | SUB15078358 Seq11 PV083099 | G | [38] |
| Hemiptera | *Adelphocoris fasciaticollis* | 17 | 1.41 | 99.85 | SUB15078358 Seq2 PV083090 | G | [39] |
| Hemiptera | *Apolygus lucorum* | 7 | 1.71 |  |  | G | [40] |
| Hemiptera | *Corythucha marmorata* | 81 | 27.93 |  |  | S^2^ | [41] |

^1^almost exclusively feeds on Asteraceae with occasional occurrences on *Chenopodium* *sp.* [42]. ^2^exlusively feeds on Asteraceae in its native range (North America). It has been accidentally introduced to Japan in 2000 [41], China in 2010 [43], and Korea in 2011 [44]. It has undergone an expansion of its host range in Asia to include plants from other families.

**Table C. Twelve herbivore species used in this study.** For insect bioassays in Experiment 1, the developmental stages of three insects are reported. For herbivory induction treatments in Experiment 3, the developmental stages and the number of individuals used in seven different herbivory treatments (H_1 to H_7) are reported. The acquisition methods of all insects are also provided: CV = commercial vendors; FC = field collection around the study site, Wuhan, in central China.

| **Orders** | **Species** | **Acquisition**  **methods** | **Experiment 1** | **Experiment 3** | | | | | | | |
| --- | --- | --- | --- | --- | --- | --- | --- | --- | --- | --- | --- |
|  |  |  | **Developmental stages** | **Developmental stages** | **H_1** | **H_2** | **H_3** | **H_4** | **H_5** | **H_6** | **H_7** |
| Lepidoptera | *Spodoptera litura* | CV | Larvae (first instar) | Larvae (second instar) | 0 | 1 | 3 | 6 | 9 | 12 | 15 |
| Lepidoptera | *Spodoptera exigua* | CV | ---- | Larvae (second instar) | 0 | 2 | 4 | 8 | 12 | 16 | 20 |
| Lepidoptera | *Helicoverpa zea* | CV | --- | Larvae (second instar) | 0 | 3 | 9 | 18 | 27 | 36 | 45 |
| Lepidoptera | *Spodoptera frugiperda* | CV | --- | Larvae (second instar) | 0 | 1 | 2 | 4 | 6 | 8 | 10 |
| Coleoptera | *Monolepta hieroglyphica* | CV | Adults | Adults | 0 | 1 | 3 | 6 | 9 | 12 | 15 |
| Coleoptera | *Luperomorpha xanthodera* | FC | --- | Adults | 0 | 1 | 3 | 6 | 9 | 12 | 15 |
| Coleoptera | *Nonarthra cyaneum* | FC | --- | Adults | 0 | 6 | 12 | 18 | 24 | 30 | 36 |
| Coleoptera | *Piazomias fausti* | FC | --- | Adults | 0 | 1 | 2 | 3 | 4 | 5 | 6 |
| Orthoptera | *Atractomorpha sinensis* | FC | Adults (only males) | Adults (only males) | 0 | 1 | 2 | 3 | 4 | 5 | 6 |
| Orthoptera | *Oecanthus rufescens* | FC | --- | Adults | 0 | 1 | 2 | 3 | 4 | 6 | 8 |
| Orthoptera | *Xenocatantops brachycerus* | FC | --- | Nymphs (~1.5cm length) | 0 | 1 | 2 | 3 | 4 | 5 | 6 |
| Orthoptera | *Phaneroptera falcata* | FC | --- | Nymphs (~1.5cm length) | 0 | 2 | 4 | 8 | 12 | 16 | 20 |

**Table D. Results of separate linear mixed models to predict the effect of six plant traits on herbivore performance.** The six traits studied, chlorogenic acid (CHA), kaempferol, rutin, trichomes, condensed tannins (tannins), and lignin, were measured in *Ambrosia artemisiifolia*. Three herbivore species, *Spodoptera litura*, *Monolepta hieroglyphica*, and *Atractomorpha sinensis*, were used in feeding bioassays. Larval weight gain was measured for *S. litura* after 5-days of feeding, and leaf areas consumed were measured for *M. hieroglyphica* and *A. sinensis* after 1-day of feeding. For each herbivore species, each plant trait was analyzed separately using a linear mixed model (LMM) across individual plants from 12 *A. artemisiifolia* populations (n = 72 for *M. hieroglyphica* and *A. sinensis*; n = 66 for *S. litura* after excluding six larval deaths), with herbivore performance as the response variable and trait levels as the fixed factor along with population as a random factor. Larval weight gain of *S. litura* and leaf area consumed by *M. hieroglyphica* were log*_e_*-transformed. The coefficient estimate for the fixed factor is presented. The statistical significance was estimated by *t*-test *p-*values, adjusted using the Benjamini–Hochberg procedure across six separate LMMs for each herbivore. Effects that were significant (adjusted *p* < 0.05) are shown in bold. *R*^2^ (M) and *R*^2^ (C) represent marginal*-* and conditional*-R*^2^, respectively.

| **Herbivores used for bioassay** | **Trait** | **Estimate** | **Standard error** | **DF** | ***t*** | ***P*** | ***R*^2^**  **(M)** | ***R*^2^**  **(C)** |
| --- | --- | --- | --- | --- | --- | --- | --- | --- |
| *Spodoptera litura* | CHA | -0.237 | 0.063 | 53 | -3.785 | **< 0.001** | 0.181 | 0.181 |
|  | Kaempferol | -0.033 | 0.008 | 53 | -4.051 | **< 0.001** | 0.202 | 0.204 |
|  | Rutin | -0.002 | 0.001 | 53 | -2.120 | **0.043** | 0.063 | 0.115 |
|  | Trichomes | -0.017 | 0.002 | 53 | -7.201 | **< 0.001** | 0.444 | 0.444 |
|  | Tannins | -0.140 | 0.068 | 53 | -2.076 | **0.043** | 0.062 | 0.095 |
|  | Lignin | -0.007 | 0.001 | 53 | -5.394 | **< 0.001** | 0.309 | 0.309 |
| *Monolepta hieroglyphica* | CHA | -0.351 | 0.087 | 59 | -4.038 | **< 0.001** | 0.187 | 0.187 |
|  | Kaempferol | -0.024 | 0.013 | 59 | -1.841 | 0.085 | 0.046 | 0.046 |
|  | Rutin | -0.002 | 0.002 | 59 | -1.351 | 0.182 | 0.025 | 0.025 |
|  | Trichomes | -0.013 | 0.005 | 59 | -2.939 | **0.009** | 0.108 | 0.108 |
|  | Tannins | -0.183 | 0.075 | 59 | -2.442 | **0.026** | 0.077 | 0.077 |
|  | Lignin | -0.007 | 0.002 | 59 | -3.840 | **< 0.001** | 0.172 | 0.172 |
| *Atractomorpha sinensis* | CHA | -1.343 | 0.297 | 59 | -4.517 | **< 0.001** | 0.223 | 0.223 |
|  | Kaempferol | -0.057 | 0.031 | 59 | -1.827 | 0.087 | 0.045 | 0.070 |
|  | Rutin | -0.013 | 0.004 | 59 | -2.930 | **0.010** | 0.107 | 0.162 |
|  | Trichomes | -0.059 | 0.016 | 59 | -3.682 | **0.002** | 0.160 | 0.160 |
|  | Tannins | -0.307 | 0.240 | 59 | -1.277 | 0.206 | 0.022 | 0.048 |
|  | Lignin | -0.011 | 0.005 | 59 | -2.159 | 0.052 | 0.057 | 0.141 |

**Table E. Results of pairwise Pearson correlation analyses among six plant traits studied in Experiment 1.** The six traits studied, chlorogenic acid (CHA), kaempferol, rutin, trichomes, condensed tannins (tannins), and lignin, were measured in *Ambrosia artemisiifolia*. Three sets of plants (n = 72 per set) were each used to evaluate the effects of these traits against one of three herbivore species, *Spodoptera litura*, *Monolepta hieroglyphica* and *Atractomorpha sinensis*. For each plant set, all possible pairwise combinations of the six traits (15 pairs in total) were analyzed using Pearson correlation. The correlation coefficient for each trait pair is reported. The statistical significance was estimated by *t*-test *p-*values, adjusted using the Benjamini–Hochberg procedure across the 15 pairwise correlations within each plant set.

| **Herbivore species**  **corresponding to plant sets** | **Paired defense traits** | ***t*** | **DF** | **r** | ***P*** |
| --- | --- | --- | --- | --- | --- |
| *Spodoptera litura* | CHA - kaempferol | 1.604 | 70 | 0.188 | 0.316 |
|  | CHA - rutin | 0.635 | 70 | 0.076 | 0.787 |
|  | CHA - trichomes | 0.413 | 70 | 0.049 | 0.787 |
|  | CHA - tannins | 0.385 | 70 | 0.046 | 0.787 |
|  | CHA - lignin | 1.640 | 70 | 0.192 | 0.316 |
|  | Kaempferol - rutin | 0.340 | 70 | 0.041 | 0.787 |
|  | Kaempferol - trichomes | 2.968 | 70 | 0.334 | 0.062 |
|  | Kaempferol - tannins | -0.653 | 70 | -0.078 | 0.787 |
|  | Kaempferol - lignin | 1.918 | 70 | 0.223 | 0.296 |
|  | Rutin - trichomes | 0.075 | 70 | 0.009 | 0.940 |
|  | Rutin - tannins | 1.299 | 70 | 0.153 | 0.425 |
|  | Rutin - lignin | 1.194 | 70 | 0.141 | 0.444 |
|  | Trichomes - tannins | 0.368 | 70 | 0.044 | 0.787 |
|  | Trichomes - lignin | 2.323 | 70 | 0.268 | 0.173 |
|  | Tannins - lignin | 1.547 | 70 | 0.182 | 0.316 |
| *Monolepta hieroglyphica* | CHA - kaempferol | -0.675 | 70 | -0.080 | 0.753 |
|  | CHA - rutin | 0.266 | 70 | 0.032 | 0.847 |
|  | CHA - trichomes | -0.799 | 70 | -0.095 | 0.753 |
|  | CHA - tannins | 0.990 | 70 | 0.117 | 0.753 |
|  | CHA - lignin | 1.536 | 70 | 0.181 | 0.753 |
|  | Kaempferol - rutin | -0.428 | 70 | -0.051 | 0.828 |
|  | Kaempferol - trichomes | 0.804 | 70 | 0.096 | 0.753 |
|  | Kaempferol - tannins | -0.728 | 70 | -0.087 | 0.753 |
|  | Kaempferol - lignin | 1.002 | 70 | 0.119 | 0.753 |
|  | Rutin - trichomes | 0.528 | 70 | 0.063 | 0.817 |
|  | Rutin - tannins | 0.364 | 70 | 0.043 | 0.828 |
|  | Rutin - lignin | 1.869 | 70 | 0.218 | 0.753 |
|  | Trichomes - tannins | 1.055 | 70 | 0.125 | 0.753 |
|  | Trichomes - lignin | 0.892 | 70 | 0.106 | 0.753 |
|  | Tannins - lignin | 0.007 | 70 | 0.001 | 0.995 |
| *Atractomorpha sinensis* | CHA - kaempferol | 0.502 | 70 | 0.060 | 0.970 |
|  | CHA - rutin | -0.037 | 70 | -0.004 | 0.970 |
|  | CHA - trichomes | 0.046 | 70 | 0.005 | 0.970 |
|  | CHA - tannins | 0.259 | 70 | 0.031 | 0.970 |
|  | CHA - lignin | 0.114 | 70 | 0.014 | 0.970 |
|  | Kaempferol - rutin | -0.267 | 70 | -0.032 | 0.970 |
|  | Kaempferol - trichomes | 0.172 | 70 | 0.021 | 0.970 |
|  | Kaempferol - tannins | 0.997 | 70 | 0.118 | 0.970 |
|  | Kaempferol - lignin | 1.837 | 70 | 0.214 | 0.528 |
|  | Rutin - trichomes | 1.247 | 70 | 0.147 | 0.970 |
|  | Rutin - tannins | 0.350 | 70 | 0.042 | 0.970 |
|  | Rutin - lignin | -0.263 | 70 | -0.031 | 0.970 |
|  | Trichomes - tannins | 1.997 | 70 | 0.232 | 0.528 |
|  | Trichomes - lignin | 0.522 | 70 | 0.062 | 0.970 |
|  | Tannins - lignin | 0.301 | 70 | 0.036 | 0.970 |

**Table F. Results of linear mixed models with multiple fixed factors to predict the effect of six plant traits on herbivore performance.** The six traits studied, chlorogenic acid (CHA), kaempferol, rutin, trichomes, condensed tannins (tannins), and lignin, were measured in *Ambrosia artemisiifolia*. Three herbivore species, Spodoptera litura, Monolepta hieroglyphica, and Atractomorpha sinensis, were used in feeding bioassays. Larval weight gain was measured for *S. litura* after 5-days of feeding, and leaf areas consumed were measured for *M. hieroglyphica* and *A. sinensis* after 1-day of feeding. Each herbivore was analyzed separately using a linear mixed model across individual plants from 12 *A. artemisiifolia* populations (n = 72 for *M. hieroglyphica* and *A. sinensis*; n = 66 for *S. litura* after excluding six larval deaths), with herbivore performance as the response variable and all six traits as fixed factors along with population as a random factor. Larval weight gain of *S. litura* and leaf area consumed by *M. hieroglyphica* were log*_e_*-transformed. All traits were standardized using Z-scores (mean = 0, SD = 1) before model fitting. The variance inflation factor (VIF) and coefficient estimate for each fixed factor are presented. The statistical significance was estimated by *t*-test *p-*values, adjusted using the Benjamini–Hochberg procedure across all tests within each model. Effects that were significant (adjusted *p* < 0.05) are shown in bold. *R*^2^ (M) and *R*^2^ (C) represent marginal*-* and conditional*-R*^2^, respectively.

| **Herbivores used for bioassay** | **Trait** | **VIF** | **Estimate** | **Standard**  **error** | **DF** | ***t*** | ***P*** | ***R*^2^**  **(M)** | ***R*^2^**  **(C)** |
| --- | --- | --- | --- | --- | --- | --- | --- | --- | --- |
| *Spodoptera*  *litura* | CHA | 1.110 | -0.088 | 0.022 | 48 | -4.055 | **< 0.001** | 0.704 | 0.704 |
|  | Kaempferol | 1.207 | -0.053 | 0.023 | 48 | -2.321 | **0.037** |  |  |
|  | Rutin | 1.046 | -0.037 | 0.021 | 48 | -1.758 | 0.085 |  |  |
|  | Trichomes | 1.311 | -0.147 | 0.024 | 48 | -6.237 | **< 0.001** |  |  |
|  | Tannins | 1.055 | -0.041 | 0.021 | 48 | -1.948 | 0.069 |  |  |
|  | Lignin | 1.260 | -0.071 | 0.023 | 48 | -3.073 | **0.007** |  |  |
| *Monolepta*  *hieroglyphica* | CHA | 1.075 | -0.170 | 0.038 | 54 | -4.429 | **< 0.001** | 0.478 | 0.478 |
|  | Kaempferol | 1.046 | -0.088 | 0.038 | 54 | -2.331 | **0.028** |  |  |
|  | Rutin | 1.060 | -0.030 | 0.038 | 54 | -0.779 | 0.439 |  |  |
|  | Trichomes | 1.056 | -0.123 | 0.038 | 54 | -3.217 | **0.007** |  |  |
|  | Tannins | 1.044 | -0.089 | 0.038 | 54 | -2.361 | **0.028** |  |  |
|  | Lignin | 1.122 | -0.116 | 0.039 | 54 | -2.944 | **0.010** |  |  |
| *Atractomorpha*  *sinensis* | CHA | 1.004 | -0.629 | 0.117 | 54 | -5.374 | **< 0.001** | 0.499 | 0.499 |
|  | Kaempferol | 1.066 | -0.207 | 0.121 | 54 | -1.715 | 0.110 |  |  |
|  | Rutin | 1.025 | -0.386 | 0.118 | 54 | -3.269 | **0.004** |  |  |
|  | Trichomes | 1.083 | -0.461 | 0.121 | 54 | -3.792 | **0.001** |  |  |
|  | Tannins | 1.072 | -0.036 | 0.121 | 54 | -0.298 | 0.767 |  |  |
|  | Lignin | 1.053 | -0.225 | 0.120 | 54 | -1.876 | 0.099 |  |  |

**Table G. Results of separate linear mixed models to predict the effect of six plant traits on plant biomass.** The six traits studied, chlorogenic acid (CHA), kaempferol, rutin, trichomes, condensed tannins (tannins), and lignin, were measured in *Ambrosia artemisiifolia*. Each trait was analyzed separately using a linear mixed model (LMM) across 1200 individual plants from all 12 *A. artemisiifolia* populations, with plant biomass as the response variable and trait levels as the fixed factor along with population as a random factor. The coefficient estimate for the fixed factor is presented. The statistical significance was estimated by *t*-test *p-*values, adjusted using the Benjamini–Hochberg procedure across six separate LMMs. Effects that were significant (adjusted *p* < 0.05) are shown in bold. *R*^2^ (M) and *R*^2^ (C) represent marginal*-* and conditional*-R*^2^, respectively.

| **Defense traits** | **Estimate** | **Standard error** | **DF** | ***t*** | ***P*** | ***R*^2^ (M)** | ***R*^2^ (C)** |
| --- | --- | --- | --- | --- | --- | --- | --- |
| CHA | 0.054 | 0.383 | 1187 | 0.142 | 0.887 | 0.000 | 0.218 |
| Kaempferol | -0.033 | 0.037 | 1187 | -0.898 | 0.444 | 0.001 | 0.218 |
| Rutin | -0.006 | 0.006 | 1187 | -1.038 | 0.444 | 0.001 | 0.219 |
| Trichomes | -0.362 | 0.020 | 1187 | -17.997 | **< 0.001** | 0.170 | 0.456 |
| Tannins | -5.406 | 0.360 | 1187 | -15.012 | **< 0.001** | 0.137 | 0.347 |
| Lignin | -0.182 | 0.011 | 1187 | -16.991 | **< 0.001** | 0.163 | 0.390 |

**Table H. Results of pairwise Pearson correlation analyses among six plant traits studied in Experiment 2.** The six traits studied, chlorogenic acid (CHA), kaempferol, rutin, trichomes, condensed tannins (tannins), and lignin, were measured in *Ambrosia artemisiifolia* plants (n = 1200) used to evaluate the growth costs of traits. All possible pairwise combinations of the six traits (15 pairs in total) were analyzed using Pearson correlation. The correlation coefficient for each trait pair is reported. The statistical significance was estimated by *t*-test *p-*values, adjusted using the Benjamini–Hochberg procedure across the 15 pairwise correlations. Effects that were significant (adjusted *p* < 0.05) are shown in bold.

| **Paired defense traits** | ***t*** | **DF** | **r** | ***P*** |
| --- | --- | --- | --- | --- |
| CHA - kaempferol | -0.892 | 1198 | -0.026 | 0.931 |
| CHA - rutin | -0.011 | 1198 | 0.000 | 0.991 |
| CHA - trichomes | -1.712 | 1198 | -0.049 | 0.327 |
| CHA - tannins | -0.979 | 1198 | -0.028 | 0.931 |
| CHA - lignin | 0.093 | 1198 | 0.003 | 0.991 |
| Kaempferol - rutin | -0.050 | 1198 | -0.001 | 0.991 |
| Kaempferol - trichomes | 0.284 | 1198 | 0.008 | 0.991 |
| Kaempferol - tannins | 0.415 | 1198 | 0.012 | 0.991 |
| Kaempferol - lignin | 0.450 | 1198 | 0.013 | 0.991 |
| Rutin - trichomes | -0.027 | 1198 | -0.001 | 0.991 |
| Rutin - tannins | 0.709 | 1198 | 0.020 | 0.991 |
| Rutin - lignin | 0.260 | 1198 | 0.008 | 0.991 |
| Trichomes - tannins | 7.568 | 1198 | 0.214 | **< 0.001** |
| Trichomes - lignin | 8.591 | 1198 | 0.241 | **< 0.001** |
| Tannins - lignin | 8.457 | 1198 | 0.237 | **< 0.001** |

**Table I. Results of a linear mixed model with multiple fixed factors to predict the effect of six plant traits on plant biomass.** The six traits studied, chlorogenic acid (CHA), kaempferol, rutin, trichomes, condensed tannins (tannins), and lignin, were measured in *Ambrosia artemisiifolia*. The analysis was conducted using a linear mixed model across 1200 individual plants from all 12 *A. artemisiifolia* populations, with plant biomass as the response variable and all six traits as fixed factors along with population as a random factor. All trait levels data were Z-score-transformed. The variance inflation factor (VIF) and coefficient estimate for each fixed factor are presented. The statistical significance was estimated by *t*-test *p-*values, adjusted using the Benjamini–Hochberg procedure across all tests. Effects that were significant (adjusted *p* < 0.05) are shown in bold. *R*^2^ (M) and *R*^2^ (C) represent marginal*-* and conditional*-R*^2^, respectively.

| **Defense traits** | **VIF** | **Estimate** | **Standard error** | **DF** | ***t*** | ***P*** | ***R*^2^ (M)** | ***R*^2^ (C)** |
| --- | --- | --- | --- | --- | --- | --- | --- | --- |
| CHA | 1.003 | -0.128 | 0.191 | 1182 | -0.672 | 0.570 | 0.325 | 0.603 |
| Kaempferol | 1.001 | -0.105 | 0.185 | 1182 | -0.568 | 0.570 |  |  |
| Rutin | 1.000 | -0.287 | 0.186 | 1182 | -1.543 | 0.185 |  |  |
| Trichomes | 1.078 | -3.057 | 0.207 | 1182 | -14.790 | **< 0.001** |  |  |
| Tannins | 1.065 | -2.384 | 0.202 | 1182 | -11.822 | **< 0.001** |  |  |
| Lignin | 1.066 | -2.824 | 0.201 | 1182 | -14.051 | **< 0.001** |  |  |

**Table J. Results of pairwise comparisons in regression slopes for traits analyzed in the linear mixed model provided in Table I.** Regression slopes were individually compared between each of chlorogenic acid (CHA), kaempferol, and rutin and each of trichomes, condensed tannins (tannins), and lignin using linear hypothesis tests. Specifically, for each pair of traits, we tested the null hypothesis that the slopes of two traits were equal by comparing a restricted model, in which the slopes of the two traits were constrained to be equal, with the original LMM model. The difference in model fit was evaluated using a chi-square test, and the significance of the slope difference was determined by the *p*-value from the chi-square test. The *p*-values were corrected across all pairwise comparisons using Benjamini–Hochberg method. Effects that were significant (adjusted *p* < 0.05) are shown in bold. The differences in slopes between each pair of traits are presented. DF represents the difference in degrees of freedom between the constrained and original models.

| **Paired defense traits** | **Differences in slopes** | **DF** | **Chi-square** | ***P*** |
| --- | --- | --- | --- | --- |
| CHA - trichomes | 2.928 | 1 | 112.330 | **< 0.001** |
| CHA - tannins | 2.256 | 1 | 65.486 | **< 0.001** |
| CHA - lignin | 2.695 | 1 | 94.991 | **< 0.001** |
| Kaempferol - trichomes | 2.951 | 1 | 111.712 | **< 0.001** |
| Kaempferol - tannins | 2.279 | 1 | 68.599 | **< 0.001** |
| Kaempferol - lignin | 2.719 | 1 | 98.143 | **< 0.001** |
| Rutin - trichomes | 2.770 | 1 | 100.652 | **< 0.001** |
| Rutin - tannins | 2.098 | 1 | 57.943 | **< 0.001** |
| Rutin - lignin | 2.537 | 1 | 86.199 | **< 0.001** |

**Table K**. **Population‑specific effects of six plant traits on plant biomass, extracted from a single multiple linear model.** The six traits studied, chlorogenic acid (CHA), kaempferol, rutin, trichomes, condensed tannins (tannins), and lignin, were measured in *Ambrosia artemisiifolia*. The analysis was conducted using a multiple linear model across 1200 individual plants from all 12 *A. artemisiifolia* populations. Plant biomass was used as the response variable, while all six traits, population and all trait × population interactions were used as explanatory variables. Adding maternal line (nested within population) as a random effect did not improve the model fit, thus this factor was removed. All trait levels data were Z-score-transformed. Coefficient estimates for each trait within each population are presented. The statistical significance was estimated by *t*-test *p-*values, adjusted using the Benjamini–Hochberg procedure across all tests. Effects that were significant (adjusted *p* < 0.05) are shown in bold.

| **Populations** | **Trait** | **Estimate** | **Standard error** | **DF** | ***t*** | ***P*** |
| --- | --- | --- | --- | --- | --- | --- |
| CD | CHA | -0.540 | 0.476 | 1116 | -1.134 | 0.441 |
| CD | Kaempferol | 1.021 | 0.614 | 1116 | 1.663 | 0.211 |
| CD | Rutin | -0.783 | 0.647 | 1116 | -1.209 | 0.410 |
| CD | Trichome | -4.199 | 0.649 | 1116 | -6.472 | **< 0.001** |
| CD | Tannin | -4.497 | 0.652 | 1116 | -6.896 | **< 0.001** |
| CD | Lignin | -4.590 | 0.639 | 1116 | -7.187 | **< 0.001** |
| DXH | CHA | -0.475 | 0.704 | 1116 | -0.675 | 0.654 |
| DXH | Kaempferol | 0.319 | 0.580 | 1116 | 0.549 | 0.722 |
| DXH | Rutin | -0.057 | 0.629 | 1116 | -0.091 | 0.982 |
| DXH | Trichome | -2.536 | 0.601 | 1116 | -4.217 | **< 0.001** |
| DXH | Tannin | -0.989 | 0.672 | 1116 | -1.471 | 0.300 |
| DXH | Lignin | -2.123 | 0.789 | 1116 | -2.691 | **0.022** |
| HP1 | CHA | 1.880 | 1.441 | 1116 | 1.305 | 0.374 |
| HP1 | Kaempferol | -0.710 | 0.607 | 1116 | -1.170 | 0.426 |
| HP1 | Rutin | 0.351 | 0.563 | 1116 | 0.625 | 0.672 |
| HP1 | Trichome | -3.786 | 0.702 | 1116 | -5.391 | **< 0.001** |
| HP1 | Tannin | -4.725 | 0.741 | 1116 | -6.379 | **< 0.001** |
| HP1 | Lignin | -2.871 | 0.698 | 1116 | -4.113 | **< 0.001** |
| HP2 | CHA | 0.396 | 0.611 | 1116 | 0.647 | 0.665 |
| HP2 | Kaempferol | 0.219 | 0.608 | 1116 | 0.359 | 0.845 |
| HP2 | Rutin | -0.164 | 0.595 | 1116 | -0.275 | 0.895 |
| HP2 | Trichome | -1.186 | 0.633 | 1116 | -1.875 | 0.142 |
| HP2 | Tannin | -1.594 | 0.592 | 1116 | -2.694 | **0.022** |
| HP2 | Lignin | -1.299 | 0.677 | 1116 | -1.918 | 0.133 |
| HP3 | CHA | -0.059 | 0.524 | 1116 | -0.112 | 0.982 |
| HP3 | Kaempferol | 0.004 | 0.683 | 1116 | 0.006 | 0.995 |
| HP3 | Rutin | -0.560 | 0.626 | 1116 | -0.893 | 0.573 |
| HP3 | Trichome | -4.952 | 0.933 | 1116 | -5.309 | **< 0.001** |
| HP3 | Tannin | -4.044 | 0.705 | 1116 | -5.737 | **< 0.001** |
| HP3 | Lignin | -3.797 | 0.659 | 1116 | -5.763 | **< 0.001** |
| JX1 | CHA | -0.057 | 0.531 | 1116 | -0.108 | 0.982 |
| JX1 | Kaempferol | -0.108 | 0.581 | 1116 | -0.186 | 0.959 |
| JX1 | Rutin | 0.011 | 0.652 | 1116 | 0.017 | 0.995 |
| JX1 | Trichome | -2.736 | 0.658 | 1116 | -4.156 | **< 0.001** |
| JX1 | Tannin | -0.410 | 0.765 | 1116 | -0.536 | 0.722 |
| JX1 | Lignin | -1.321 | 0.686 | 1116 | -1.924 | 0.133 |
| JX2 | CHA | -0.567 | 0.679 | 1116 | -0.834 | 0.582 |
| JX2 | Kaempferol | -0.566 | 0.622 | 1116 | -0.910 | 0.573 |
| JX2 | Rutin | -0.793 | 0.657 | 1116 | -1.207 | 0.410 |
| JX2 | Trichome | -4.847 | 1.222 | 1116 | -3.965 | **< 0.001** |
| JX2 | Tannin | -1.831 | 0.750 | 1116 | -2.442 | **0.039** |
| JX2 | Lignin | -2.811 | 0.749 | 1116 | -3.751 | **< 0.001** |
| JX3 | CHA | -0.048 | 0.869 | 1116 | -0.055 | 0.983 |
| JX3 | Kaempferol | -0.513 | 0.597 | 1116 | -0.860 | 0.573 |
| JX3 | Rutin | -0.070 | 0.614 | 1116 | -0.114 | 0.982 |
| JX3 | Trichome | -3.171 | 0.626 | 1116 | -5.064 | **< 0.001** |
| JX3 | Tannin | -0.453 | 0.651 | 1116 | -0.696 | 0.649 |
| JX3 | Lignin | -0.977 | 0.690 | 1116 | -1.416 | 0.323 |
| JX4 | CHA | -1.009 | 1.115 | 1116 | -0.905 | 0.573 |
| JX4 | Kaempferol | 0.256 | 0.733 | 1116 | 0.349 | 0.845 |
| JX4 | Rutin | 0.595 | 0.582 | 1116 | 1.021 | 0.515 |
| JX4 | Trichome | -3.922 | 0.580 | 1116 | -6.761 | **< 0.001** |
| JX4 | Tannin | -1.898 | 0.762 | 1116 | -2.491 | **0.036** |
| JX4 | Lignin | -2.931 | 0.689 | 1116 | -4.253 | **< 0.001** |
| WN | CHA | -0.038 | 0.614 | 1116 | -0.062 | 0.983 |
| WN | Kaempferol | -0.439 | 0.587 | 1116 | -0.748 | 0.624 |
| WN | Rutin | 0.234 | 0.632 | 1116 | 0.370 | 0.845 |
| WN | Trichome | -4.348 | 0.737 | 1116 | -5.902 | **< 0.001** |
| WN | Tannin | -1.205 | 0.680 | 1116 | -1.773 | 0.172 |
| WN | Lignin | -1.363 | 0.634 | 1116 | -2.148 | 0.082 |
| XZ1 | CHA | 0.397 | 0.490 | 1116 | 0.810 | 0.591 |
| XZ1 | Kaempferol | 0.458 | 0.618 | 1116 | 0.740 | 0.624 |
| XZ1 | Rutin | -0.591 | 0.665 | 1116 | -0.889 | 0.573 |
| XZ1 | Trichome | -2.412 | 0.801 | 1116 | -3.012 | **0.009** |
| XZ1 | Tannin | -1.554 | 0.609 | 1116 | -2.551 | **0.031** |
| XZ1 | Lignin | -2.414 | 0.644 | 1116 | -3.747 | **< 0.001** |
| XZ2 | CHA | 0.851 | 0.984 | 1116 | 0.865 | 0.573 |
| XZ2 | Kaempferol | -0.779 | 0.642 | 1116 | -1.213 | 0.410 |
| XZ2 | Rutin | -0.872 | 0.666 | 1116 | -1.310 | 0.374 |
| XZ2 | Trichome | -2.157 | 0.698 | 1116 | -3.091 | **0.007** |
| XZ2 | Tannin | -3.815 | 0.692 | 1116 | -5.515 | **< 0.001** |
| XZ2 | Lignin | -5.173 | 0.628 | 1116 | -8.234 | **< 0.001** |

**Table L.** **Results of regression analyses for herbivory intensity on the levels of six plant traits.** Twelve insect species were used to impose herbivory treatments on *Ambrosia artemisiifolia*. For each herbivore treatment, the six plant traits studied, chlorogenic acid (CHA), kaempferol, rutin, trichomes, condensed tannins (tannins), and lignin, were analyzed separately. Trichome densities were analyzed using quasi-Poisson models and other traits were analyzed using Gaussian models. Herbivory intensity (the percentage of leaf area damaged) was included as a linear or segmented term with a single breakpoint under a null-left-slope constraint. The model with the best fit was identified based on a combination of Davies test and Akaike Information Criterion (AIC) or quasi-AIC (QAIC). Coefficient estimates [Linear models: slopes for the whole damage range; Segmented models: break points with 95% confidence intervals (CIs) and slopes after break points] are presented. The goodness of fit for Gaussian models are reported as *R*^2^ and that for quasi-Poisson models are reported as explained deviances: [1 – (residual deviance/null deviance)]. The statistical significance of the slopes was estimated by CIs from *t* tests. Across all analyses for each herbivore, CIs were corrected for multiple comparisons based on the number of tests and the number of significant results at a false discovery rate threshold of 0.05. Significant effects (*i.e.*, adjusted 95% CIs not crossing zero) are shown in bold. SE = standard errors for the regression slopes.

| **Herbivores** | **Traits** | ***P-*values from Davies test** | **AIC or QAIC for Linear models** | **AIC or QAIC for segmented models** | **Selected models** | ***R*^2^ or explained deviances** | **Break points**  **(95% CIs, %)** | **Slopes** | **SE** | ***t*** | **Adjusted 95% CIs** |
| --- | --- | --- | --- | --- | --- | --- | --- | --- | --- | --- | --- |
| *Spodoptera* | CHA | 1.000 | 301.828 | 302.943 | linear | 0.339 | ------ | 2.181 | 0.274 | 7.974 | **1.640, 2.723** |
| *litura* | Kaempferol | 0.223 | 834.294 | 836.294 | linear | 0.565 | ------ | 28.720 | 2.263 | 12.692 | **24.241, 33.198** |
|  | Rutin | 0.177 | 1370.874 | 1372.259 | linear | 0.481 | ------ | 203.772 | 19.027 | 10.710 | **166.112, 241.432** |
|  | Trichomes | **< 0.001** | 299.396 | 268.522 | segmented | 0.421 | 48.9 (41.0-56.8) | 1.965 | 0.283 | 6.932 | **1.409, 2.520** |
|  | Tannins | **< 0.001** | 299.633 | 273.189 | segmented | 0.490 | 45.4 (38.6-52.2) | 6.050 | 0.832 | 7.272 | **4.403, 7.697** |
|  | Lignin | **0.005** | 1221.720 | 1210.249 | segmented | 0.290 | 36.6 (25.1-48.0) | 127.483 | 25.219 | 5.055 | **77.563, 177.400** |
| *Spodoptera* | CHA | 0.651 | 233.824 | 235.824 | linear | 0.101 | ------ | 0.748 | 0.200 | 3.742 | **0.318, 1.179** |
| *exigua* | Kaempferol | 0.889 | 820.592 | 822.528 | linear | 0.024 | ------ | 3.607 | 2.053 | 1.757 | -0.811, 8.024 |
|  | Rutin | 0.647 | 1346.184 | 1348.161 | linear | 0.447 | ------ | 165.413 | 16.525 | 10.010 | **129.851, 200.975** |
|  | Trichomes | **0.003** | 305.943 | 293.826 | segmented | 0.245 | 52.5 (39.2-65.9) | 1.363 | 0.389 | 3.503 | **0.535, 2.190** |
|  | Tannins | 0.511 | 260.899 | 261.008 | linear | 0.000 | ------ | -0.009 | 0.223 | -0.038 | -0.488, 0.471 |
|  | Lignin | **< 0.001** | 1227.279 | 1213.289 | segmented | 0.405 | 34.2 (22.2-46.2) | 145.170 | 26.032 | 5.577 | **89.142, 201.200** |
| *Helicoverpa* | CHA | 0.444 | 295.783 | 297.377 | linear | 0.330 | ------ | 1.982 | 0.254 | 7.808 | **1.436, 2.528** |
| *zea* | Kaempferol | 0.308 | 788.496 | 790.496 | linear | 0.025 | ------ | 3.198 | 1.793 | 1.784 | -0.660, 7.057 |
|  | Rutin | 0.340 | 1412.469 | 1414.469 | linear | 0.429 | ------ | 205.797 | 21.328 | 9.649 | **159.898, 251.696** |
|  | Trichomes | **< 0.001** | 296.718 | 282.278 | segmented | 0.281 | 48.6 (37.1-60.1) | 1.299 | 0.268 | 4.851 | **0.729, 1.869** |
|  | Tannins | **< 0.001** | 370.380 | 323.090 | segmented | 0.616 | 43.8 (37.7-50.0) | 8.179 | 0.905 | 9.038 | **6.231, 10.126** |
|  | Lignin | 0.116 | 1153.188 | 1153.116 | linear | 0.001 | ------ | 1.976 | 7.623 | 0.259 | -14.428, 18.381 |
| *Spodoptera* | CHA | 0.754 | 201.319 | 203.178 | linear | 0.011 | ------ | 0.193 | 0.167 | 1.154 | -0.151, 0.538 |
| *frugiperda* | Kaempferol | 0.328 | 846.301 | 848.301 | linear | 0.252 | ------ | 13.992 | 2.164 | 6.467 | **9.539, 18.446** |
|  | Rutin | 0.221 | 1341.033 | 1343.033 | linear | 0.220 | ------ | 91.138 | 15.411 | 5.914 | **59.420, 122.856** |
|  | Trichomes | **0.020** | 270.341 | 263.270 | segmented | 0.212 | 45.0 (29.6-60.5) | 1.000 | 0.260 | 3.850 | **0.471, 1.529** |
|  | Tannins | **< 0.001** | 306.009 | 269.998 | segmented | 0.609 | 45.8 (39.6-51.9) | 6.441 | 0.730 | 8.822 | **4.938, 7.943** |
|  | Lignin | **0.046** | 1217.911 | 1211.946 | segmented | 0.402 | 32.4 (20.5-44.2) | 128.591 | 21.030 | 6.115 | **85.303, 171.880** |
| *Monolepta* | CHA | 0.804 | 325.003 | 326.295 | linear | 0.507 | ------ | 2.870 | 0.254 | 11.284 | **2.347, 3.393** |
| *hieroglyphica* | Kaempferol | 0.785 | 812.764 | 814.764 | linear | 0.416 | ------ | 16.552 | 1.762 | 9.393 | **12.925, 20.178** |
|  | Rutin | 0.801 | 1267.622 | 1269.573 | linear | 0.007 | ------ | 10.171 | 10.713 | 0.949 | -11.879, 32.221 |
|  | Trichomes | **0.002** | 295.507 | 285.537 | segmented | 0.316 | 35.2 (18.9-51.5) | 0.837 | 0.164 | 5.115 | **0.504, 1.170** |
|  | Tannins | **< 0.001** | 264.638 | 247.528 | segmented | 0.397 | 44.7 (33.3-56.1) | 3.276 | 0.579 | 5.655 | **2.084, 4.469** |
|  | Lignin | **< 0.001** | 1219.830 | 1204.885 | segmented | 0.609 | 31.0 (22.0-40.1) | 169.20 | 18.751 | 9.024 | **130.600, 207.800** |
| *Luperomorpha* | CHA | 1.000 | 294.114 | 295.942 | linear | 0.227 | ------ | 1.625 | 0.269 | 6.038 | **1.071, 2.179** |
| *xanthodera* | Kaempferol | 0.791 | 811.804 | 813.804 | linear | 0.226 | ------ | 12.650 | 2.100 | 6.025 | **8.329, 16.972** |
|  | Rutin | 0.230 | 1266.485 | 1268.320 | linear | 0.019 | ------ | 19.547 | 12.757 | 1.532 | -6.709, 45.802 |
|  | Trichomes | **0.017** | 293.077 | 285.779 | segmented | 0.283 | 41.5 (28.4-54.7) | 1.253 | 0.275 | 4.564 | **0.694, 1.812** |
|  | Tannins | **< 0.001** | 298.254 | 284.242 | segmented | 0.491 | 29.4 (20.1-38.6) | 4.410 | 0.617 | 7.149 | **3.140, 5.680** |
|  | Lignin | **< 0.001** | 1210.144 | 1191.846 | segmented | 0.431 | 30.2 (19.6-40.9) | 146.364 | 23.716 | 6.171 | **97.547, 195.180** |
| *Nonarthra* | CHA | 0.732 | 274.209 | 276.063 | linear | 0.268 | ------ | 1.875 | 0.278 | 6.740 | **1.244, 2.507** |
| *cyaneum* | Kaempferol | 0.839 | 774.983 | 776.983 | linear | 0.005 | ------ | 1.608 | 2.030 | 0.792 | -2.998, 6.214 |
|  | Rutin | 0.524 | 1278.123 | 1280.123 | linear | 0.028 | ------ | 28.038 | 14.948 | 1.876 | -5.879, 61.955 |
|  | Trichomes | **< 0.001** | 320.428 | 298.430 | segmented | 0.412 | 37.6 (30-45.3) | 1.576 | 0.217 | 7.252 | **1.089, 2.063** |
|  | Tannins | 0.339 | 260.006 | 259.335 | linear | 0.010 | ------ | 0.288 | 0.263 | 1.096 | -0.309, 0.885 |
|  | Lignin | **< 0.001** | 1181.582 | 1165.722 | segmented | 0.229 | 44.4 (35.3-53.6) | 145.784 | 32.776 | 4.448 | **71.409, 220.160** |
| *Piazomias* | CHA | 0.276 | 298.565 | 300.497 | linear | 0.200 | ------ | 1.378 | 0.248 | 5.560 | **0.844, 1.911** |
| *fausti* | Kaempferol | 0.300 | 802.250 | 803.945 | linear | 0.474 | ------ | 19.329 | 1.829 | 10.570 | **15.393, 23.264** |
|  | Rutin | 0.430 | 1261.005 | 1262.339 | linear | 0.004 | ------ | 8.253 | 11.291 | 0.731 | -16.046, 32.552 |
|  | Trichomes | **0.006** | 269.728 | 262.296 | segmented | 0.278 | 36.2 (20.2-52.2) | 0.846 | 0.173 | 4.877 | **0.477, 1.215** |
|  | Tannins | **< 0.001** | 333.794 | 315.532 | segmented | 0.541 | 33.0 (24.5-41.6) | 4.686 | 0.529 | 8.853 | **3.547, 5.825** |
|  | Lignin | 0.109 | 1200.903 | 1200.923 | linear | 0.024 | ------ | 8.983 | 8.895 | 1.010 | -10.160, 28.126 |
| *Atractomorpha* | CHA | 0.359 | 270.547 | 272.547 | linear | 0.278 | ------ | 1.619 | 0.234 | 6.907 | **1.136, 2.101** |
| *sinensis* | Kaempferol | 0.304 | 817.404 | 819.404 | linear | 0.452 | ------ | 20.755 | 2.053 | 10.111 | **16.530, 24.980** |
|  | Rutin | 0.676 | 1361.307 | 1363.307 | linear | 0.420 | ------ | 168.228 | 17.769 | 9.467 | **131.656, 204.800** |
|  | Trichomes | **0.001** | 289.618 | 276.637 | segmented | 0.348 | 42.2 (31.0-53.5) | 1.226 | 0.213 | 5.757 | **0.792, 1.660** |
|  | Tannins | 0.551 | 257.494 | 259.121 | linear | 0.011 | ------ | 0.222 | 0.223 | 0.996 | -0.236, 0.680 |
|  | Lignin | **0.003** | 1198.455 | 1187.479 | segmented | 0.504 | 31.4 (22.3-40.5) | 144.416 | 18.221 | 7.926 | **106.910, 181.920** |
| *Oecanthus* | CHA | 0.881 | 289.113 | 291.113 | linear | 0.240 | ------ | 1.614 | 0.258 | 6.263 | **1.059, 2.169** |
| *rufescens* | Kaempferol | 0.901 | 842.738 | 844.738 | linear | 0.411 | ------ | 21.571 | 2.319 | 9.303 | **16.581, 26.561** |
|  | Rutin | 0.776 | 1295.700 | 1296.847 | linear | 0.023 | ------ | 23.749 | 13.992 | 1.697 | -6.361, 53.860 |
|  | Trichomes | **0.002** | 308.143 | 296.384 | segmented | 0.361 | 39.6 (27.8-51.4) | 1.139 | 0.206 | 5.537 | **0.701, 1.577** |
|  | Tannins | **0.020** | 262.318 | 255.731 | segmented | 0.260 | 29.8 (14.4-45.2) | 2.118 | 0.456 | 4.647 | **1.137, 3.099** |
|  | Lignin | 0.620 | 1204.521 | 1206.249 | linear | 0.002 | ------ | -4.494 | 9.744 | -0.461 | -25.463, 16.475 |
| *Xenocatantops* | CHA | 0.708 | 255.016 | 257.016 | linear | 0.183 | ------ | 1.101 | 0.209 | 5.271 | **0.651, 1.550** |
| *brachycerus* | Kaempferol | 0.228 | 772.524 | 771.193 | linear | 0.004 | ------ | 1.214 | 1.628 | 0.745 | -2.290, 4.718 |
|  | Rutin | 0.883 | 1385.039 | 1387.039 | linear | 0.445 | ------ | 184.659 | 18.508 | 9.977 | **144.830, 224.488** |
|  | Trichomes | **0.001** | 307.916 | 294.952 | segmented | 0.335 | 44.8 (31.8-57.9) | 1.069 | 0.200 | 5.340 | **0.643, 1.494** |
|  | Tannins | 0.403 | 259.719 | 260.329 | linear | 0.000 | ------ | 0.062 | 0.213 | 0.291 | -0.396, 0.520 |
|  | Lignin | **0.041** | 1221.494 | 1216.489 | segmented | 0.295 | 34.8 (20.3-49.2) | 102.462 | 20.197 | 5.073 | **58.993, 145.930** |
| *Phaneroptera* | CHA | 0.261 | 212.689 | 214.651 | linear | 0.026 | ------ | 0.313 | 0.171 | 1.824 | -0.040, 0.665 |
| *falcata* | Kaempferol | 1.000 | 814.192 | 816.157 | linear | 0.371 | ------ | 15.953 | 1.865 | 8.556 | **12.115, 19.791** |
|  | Rutin | 0.195 | 1308.455 | 1310.455 | linear | 0.207 | ------ | 75.512 | 13.256 | 5.697 | **48.229, 102.794** |
|  | Trichomes | **0.011** | 305.956 | 296.822 | segmented | 0.304 | 47.0 (34.0-60.0) | 1.091 | 0.211 | 5.181 | **0.662, 1.520** |
|  | Tannins | **< 0.001** | 293.586 | 261.253 | segmented | 0.545 | 43.2 (34.9-51.4) | 4.642 | 0.585 | 7.937 | **3.438, 5.846** |
|  | Lignin | **0.015** | 1247.262 | 1239.937 | segmented | 0.488 | 31.2 (20.4-42.0) | 157.295 | 21.421 | 7.343 | **113.200, 201.390** |

**Table M. Regression slope comparisons in linear mixed models with multiple fixed factors predicting effects of six plant traits on herbivore performance.** The six traits studied, chlorogenic acid (CHA), kaempferol, rutin, trichomes, condensed tannins (tannins), and lignin, were measured in *Ambrosia artemisiifolia*. Three herbivore species, Spodoptera litura, Monolepta hieroglyphica, and Atractomorpha sinensis were used in feeding bioassays. The data and analytical procedures were identical to those used in Table F. Traits were classified into two groups based on their induction patterns in response to increasing herbivore damage in Experiment 3: group 1 exhibited a linear response, while group 2 showed a segmented response. Traits not induced in Experiment 3 were excluded from subsequent analyses. Within each model, the average regression slopes of the two groups were compared using linear hypothesis tests. Specifically, we tested the null hypothesis that the slopes of the two groups were equal by comparing a restricted model, in which the slopes of the two groups were constrained to be equal, with the original model. The difference in model fit was evaluated using a chi-square test, and the significance of the slope difference was determined by the *p*-value from the chi-square test. The differences in average slopes between the two groups of traits are presented. DF represents the differences in degrees of freedom between the constrained and original models.

| **Herbivores used for bioassay** | **Groups of traits** | **Differences in average slopes** | **DF** | **Chi-square** | ***P*** |
| --- | --- | --- | --- | --- | --- |
| *Spodoptera litura* | Group 1 (CHA, Kaempferol, Rutin)  -  Group 2 (Trichomes, Tannins, Lignin) | 0.027 | 1 | 2.405 | 0.121 |
| *Monolepta hieroglyphica* | Group 1 (CHA, Kaempferol)  -  Group 2 (Trichomes, Tannins, Lignin) | -0.020 | 1 | 0.596 | 0.440 |
| *Atractomorpha sinensis* | Group 1 (CHA, Kaempferol, Rutin)  -  Group 2 (Trichomes, Lignin) | -0.065 | 1 | 3.707 | 0.054 |

**References**

1. Folmer O, Black M, Hoeh W, Lutz R, Vrijenhoek R. DNA primers for amplification of mitochondrial cytochrome c oxidase subunit I from diverse metazoan invertebrates. Mol Mar Biol Biotechnol. 1994;3: 294–299.

2. Wan J, Huang B, Yu H, Peng S. Reassociation of an invasive plant with its specialist herbivore provides a test of the shifting defence hypothesis. J Ecol. 2019;107: 361–371. doi:10.1111/1365-2745.13019

3. Wang Y, Siemann E, Wheeler GS, Zhu L, Gu X, Ding J. Genetic variation in anti-herbivore chemical defences in an invasive plant. J Ecol. 2012;100: 894–904. doi:10.1111/j.1365-2745.2012.01980.x

4. Broadhurst RB, Jones WT. Analysis of condensed tannins using acidified vanillin. J Sci Food Agric. 1978;29: 788–794. doi:10.1002/jsfa.2740290908

5. Fukushima RS, Kerley MS. Use of lignin extracted from different plant sources as standards in the spectrophotometric acetyl bromide lignin method. J Agric Food Chem. 2011;59: 3505–3509. doi:10.1021/jf104826n

6. Moreira-Vilar FC, Siqueira-Soares R de C, Finger-Teixeira A, Oliveira DM de, Ferro AP, da Rocha GJ, et al. The acetyl bromide method is faster, simpler and presents best recovery of lignin in different herbaceous tissues than klason and thioglycolic acid methods. Plos One. 2014;9: e110000. doi:10.1371/journal.pone.0110000

7. Yin W, Zhou L, Yang K, Fang J, Biere A, Callaway RM, et al. Rapid evolutionary trade-offs between resistance to herbivory and tolerance to abiotic stress in an invasive plant. Ecol Lett. 2023;26: 942–954. doi:10.1111/ele.14221

8. Benjamini Y, Hochberg Y. Controlling the false discovery rate: a practical and powerful approach to multiple testing. J R Stat Soc Ser B. 1995;57: 289–300. doi:10.1111/j.2517-6161.1995.tb02031.x

9. Nakagawa S, Schielzeth H. A general and simple method for obtaining *R*^2^ from generalized linear mixed-effects models. Methods Ecol Evol. 2013;4: 133–142. doi:10.1111/j.2041-210x.2012.00261.x

10. Benjamini Y, Yekutieli D. False discovery rate–adjusted multiple confidence intervals for selected parameters. J Am Stat Assoc. 2005;100: 71–81. doi:10.1198/016214504000001907

11. Muggeo VMR. Testing with a nuisance parameter present only under the alternative: a score-based approach with application to segmented modelling. J Stat Comput Simul. 2016;86: 3059–3067. doi:10.1080/00949655.2016.1149855

12. R Core Team. R: A language and environment for statistical computing. Vienna, Austria: R Foundation for Statistical Computing; 2024.

13. Pinheiro J, Bates D, R Core Team. nlme: linear and nonlinear mixed effects models. 2024. Available: https://cran.r-project.org/web/packages/nlme/index.html

14. Muggeo VMR. Segmented: an R package to fit regression models with broken-line relationships. R News. 2008;8: 20–25. Available: https://cran.r-project.org/doc/Rnews/

15. Bartoń K. MuMIn: Multi-Model Inference. 2024. Available: https://cran.r-project.org/web/packages/MuMIn/index.html

16. Bolker B, R Development Core Team, Giné-Vázquez I. bbmle: tools for general maximum likelihood estimation. 2023. Available: https://cran.r-project.org/web/packages/bbmle/index.html

17. Fox J, Weisberg S. An {R} Companion to Applied Regression. Thousand Oaks, CA: Sage; 2019.

18. Zahiri R, Lafontaine D, Schmidt C, Holloway JD, Kitching IJ, Mutanen M, et al. Relationships among the basal lineages of Noctuidae (Lepidoptera, Noctuoidea) based on eight gene regions. Zool Scr. 2013;42: 488–507. doi:10.1111/zsc.12022

19. Krinski D, Godoy AF. First record of *Helicoverpa armigera* (Lepidoptera: Noctuidae) feeding on *Plectranthus neochilus* (Lamiales: Lamiaceae) in Brazil. Fla Entomol. 2015;98: 1238–1240. doi:10.1653/024.098.0434

20. Ahmad M, Ghaffar A, Rafiq M. Host plants of leaf worm, *Spodoptera litura* (Fabricius) (Lepidoptera: Noctuidae) in Pakistan. Asian J Agric Biol. 2013;1: 23–28.

21. Ma J, Wan F, Guo J, You L, Yang H. Ecological adaptability and application safety of *Epiblema strenuana* as a biocontrol agent against ragweed. J Appl Ecol. 2003;14: 1391–1394 (in Chinese with English abstract).

22. Sady EA, Kiełkiewicz M, Kozłowski MW. The rose flea beetle (*Luperomorpha xanthodera*), Coleoptera: Chrysomelidae), an alien species in central Poland−from an episodic occurrence in an established population. J Plant Prot Res. 2020;60: 86–97. doi:10.24425/jppr.2020.132209

23. Gao Y, Xu W, Shi SS, Cui J, Xu B. Catalogue of host plants of *Monolepta hieroglyphica* (Motschulsky). Hubei Agric Sci. 2017;56: 865–869 (in Chinese with English abstract).

24. Yu P, Wang S. Alticine beetles of Mt. Mogan (Coleoptera: Chrysomelidae). J Zhejiang For Coll. 1992;9: 489–490 (in Chinese with English abstract).

25. Augustinus BA, Gentili R, Horvath D, Naderi R, Sun Y, Tournet A-MTE, et al. Assessing the risks of non-target feeding by the accidentally introduced ragweed leaf beetle, *Ophraella communa*, to native European plant species. Biol Control. 2020;150: 104356. doi:10.1016/j.biocontrol.2020.104356

26. Palaniswamy P, Lamb RJ. Host preferences of the flea beetles *Phyllotreta cruciferae* and *P. striolata* (Coleoptera: Chrysomelidae) for Crucifer Seedlings. J Econ Entomol. 1992;85: 743–752. doi:10.1093/jee/85.3.743

27. Zheng H, Wu Y, Ding J, Binion D, Fu W, Reardon R. Invasive plants established in the United States that are found in Asia and their associated natural enemies. Morgantown, West Virginia, USA.: Forest Health Technology Enterprise Team; 2005.

28. Tian F. Preliminary observation of the biological characteristics of *Acrida cinerea*. Plant Prot. 2009;35: 147–148 (in Chinese with English abstract).

29. Chand DS, Nandy S. Biology of *Aiolopus thalassinus tamulus* (Fabricius) (Orthoptera: Acrididae) on a salt tolerant rice variety Dudheswar (*Oryza sativa* Linn.) of Sunderban, West Bengal. J Appl Zool Res. 2016;27: 201–205.

30. Ma G, Gao J, Lian M. Identification of glutathione S-transferase (GST) gene family from *Xenocatantops humilis brachycerus* and its insecticide sensitivity. J Northwest F Univ. 2020;48: 99–105 (in Chinese with English abstract).

31. Zhang H, Zhang X, Li Q. On the pattern of occurrence of *Chondracris rosea* (De Geer) and techniques of its control. Entomol Knowl. 1993;30: 12–14 (in Chinese).

32. Pu G, Mao J. List of mulberry pests in Anhui province. China Seric. 2013;34: 17–24 (in Chinese). doi:10.16839/j.cnki.zgcy.2013.01.005

33. Li W, Gao Y, Cui J, Shi S. Effects of temperature on the development and fecundity of *Atractomorpha sinensis* (Orthoptera: Pyrgomorphidae). J Econ Entomol. 2020;113: 2530–2539. doi:10.1093/jee/toaa145

34. Junker RR. Scents as floral defence: impact on species and communities, mechanisms and ecological consequences. University of Würzburg. 2010.

35. Singh AP. Insect pests of Western Himalayan oaks in uttarakhand. Dehradun, India: Forest Research Institute; 2021.

36. Dong Y, Shen D, Dou D, Xia A. Characterization of salivary secreted proteins that induce cell death from *Riptortus pedestris* (Fabricius) and their roles in insect-plant interactions. Front Plant Sci. 2022;13: 912603. doi:10.3389/fpls.2022.912603

37. Singh G, Singh NP, Singh R. Food plants of a major agricultural pest *Aphis gossypii Glover* (Homoptera: Aphididae) from India: an updated checklist. Int J Life Sci Biotechnol Pharma Res. 2014;3: 1.

38. Patel NM, Patel N, Raghunandan BL. First host record of false chinch bug, *Nysius inconspicuus* (Hemiptera: Lygaeidae) on sesame in Anand (Gujarat, India). Insect Environ. 2022;25: 3.

39. Zhang L, Cai W, Luo J, Zhang S, Li W, Wang C, et al. Population genetic structure and expansion patterns of the cotton pest *Adelphocoris fasciaticollis*. J Pest Sci. 2018;91: 539–550. doi:10.1007/s10340-017-0939-8

40. Lu Y, Wu K, Wyckhuys KAG, Guo Y. Overwintering hosts of *Apolygus lucorum* (Hemiptera: Miridae) in northern China. Crop Prot. 2010;29: 1026–1033. doi:10.1016/j.cropro.2010.03.017

41. Tsukada M, Hasegawa S. Host range expansion by the invasive herbivore *Corythucha marmorata* (Uhler, 1878) is not caused by better quality of new hosts. J Entomol Res Soc. 2021;23: 1–10. doi:10.51963/jers.v23i1.1846

42. McClay AS. Observations on the biology and host specificity of *Epiblema strenuana* [Lepidoptera, Tortricidae], a potential biocontrol agent for *Parthenium hysterophorus* [Compositae]. Entomophaga. 1987;32: 23–34. doi:10.1007/BF02390928

43. Zhu J. Distribution and hazards of new record *Corythucha marmorata* (Uhler, 1878) in Shanghai. J Anhui Agric Sci. 2018;46: 153–156 (in Chinese with English abstract).

44. Kim DE, Kil J. Geographical distribution and host plants of *Corythucha marmorata* (Uhler) (Hemiptera: Tingidae) in Korea. Korean J Appl Entomol. 2014;53: 185–191. doi:10.5656/KSAE.2013.11.0.073
