# Supplementary figures and images for: Plants respond to herbivory through sequential induction of cheaper defenses before more costly ones
Source: PLoS Biol. 2025 Aug 14;23(8):e3003280. doi: 10.1371/journal.pbio.3003280 (PMC12352644; doi:10.1371/journal.pbio.3003280)

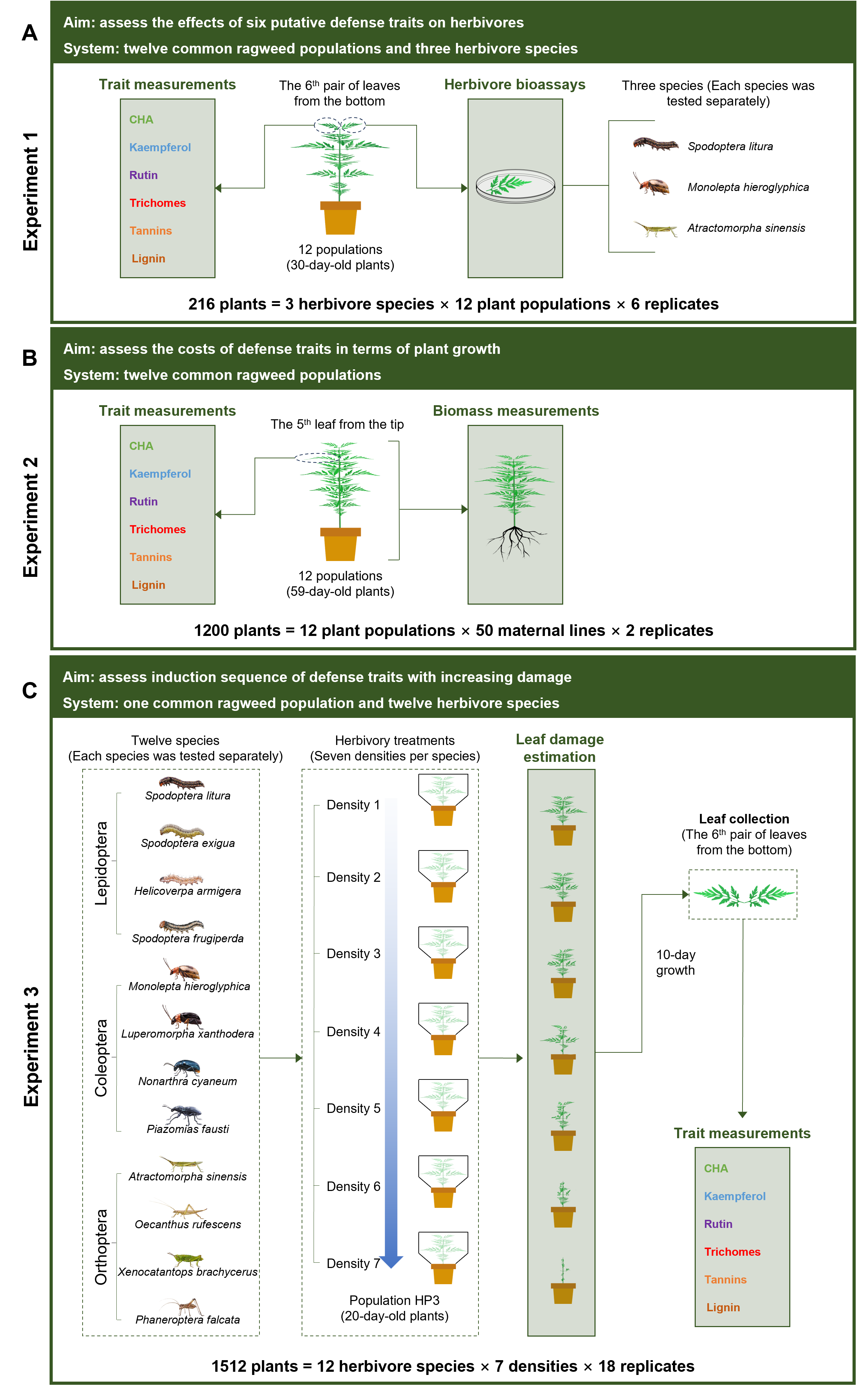

Supplement: S1 Fig — Experiment 1 (A) assessed the defense efficacy of six putative defense traits. We collected the sixth leaf pair (from the bottom) of 30-day-old plants: one leaf was used to quantify six putative defense traits, and the other to assess resistance to herbivory. This procedure was repeated three times, each with a different insect herbivore species. Experiment 2 (B) assessed the growth costs of six defense traits. We collected the fifth leaf (from the tip) of 59-day-old plants to measure six defense traits, then harvested, dried and weighed the rest (including above- and belowground parts). Experiment 3 (C) assessed induction sequence of defense traits with increasing herbivory. We exposed 20-day-old plants to each of 12 generalist herbivore species at seven increasing density levels (Density 1–7). After 2 days of feeding, insects were removed, and damage severity was recorded. After 10 days of growth, we collected the sixth leaf pair (from the bottom) for defense‐trait measurements. Information on plant materials and herbivore species is provided in Tables A and B in S1 File, respectively. (TIF) [file pbio.3003280.s002.tif]

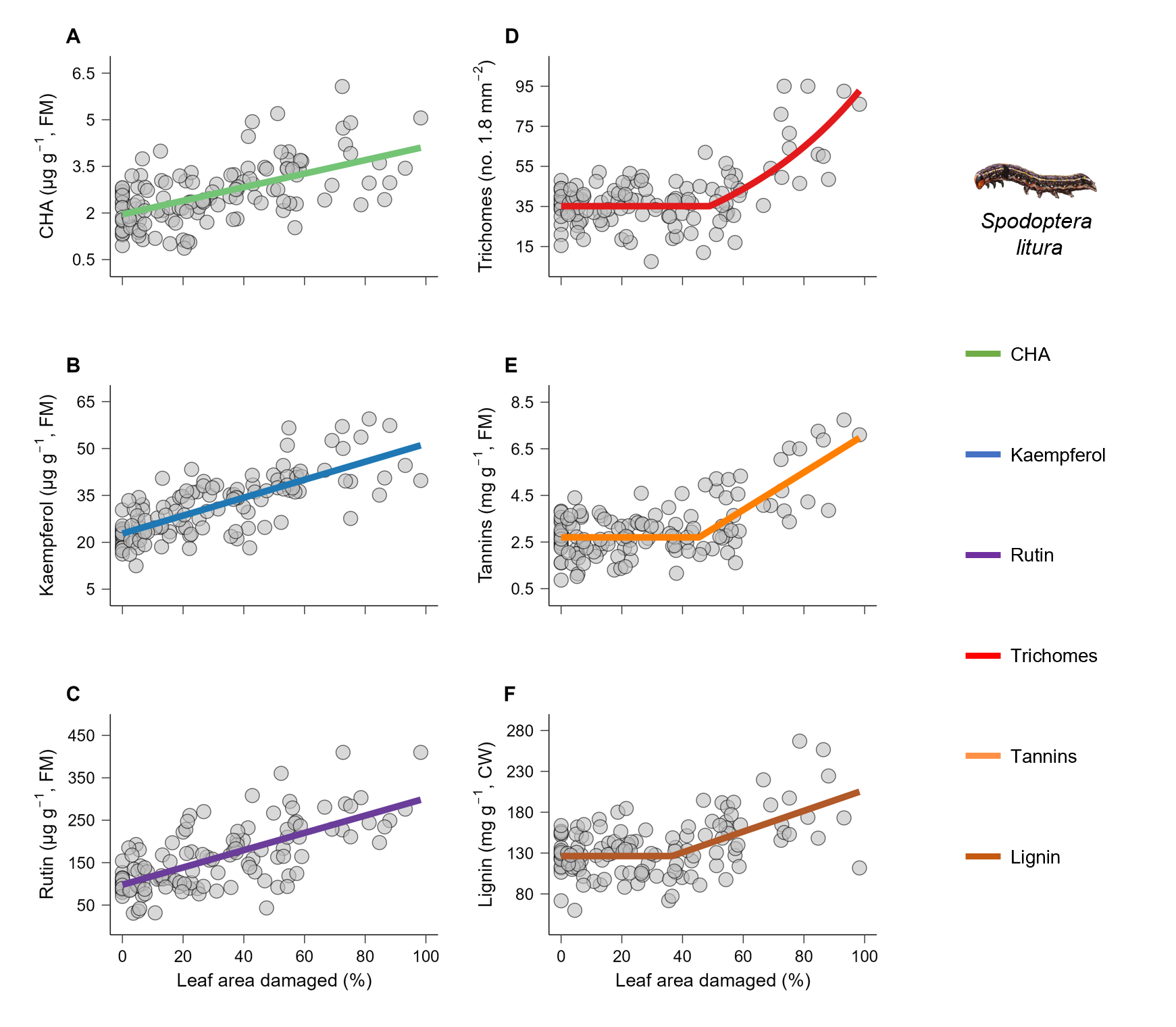

Supplement: S2 Fig — Relationships between levels of each of six traits, chlorogenic acid (CHA), kaempferol, rutin, trichomes, condensed tannins (tannins), and lignin, in Ambrosia artemisiifolia and the percentage of leaf area damaged by S. litura. Each trait was analyzed separately. The reaction norm for each trait was chosen between a linear or segmented model based on a combination of the Davies test and AIC/QAIC. Data points represent individual replicates (n = 18 per density treatment, seven density treatments). The different line colors represent different traits. Solid lines indicate significant effects (adjusted 95% confidence intervals not crossing zero) of herbivore damage on levels of traits. Predicted trichome densities are presented on the original scale (i.e., # per 1.8 mm2). Statistical results are in Table L in S1 File. FM, leaf fresh biomass. CW, cell wall. The data underlying this figure can be found in https://doi.org/10.6084/m9.figshare.29364695. (TIF) [file pbio.3003280.s003.tif]

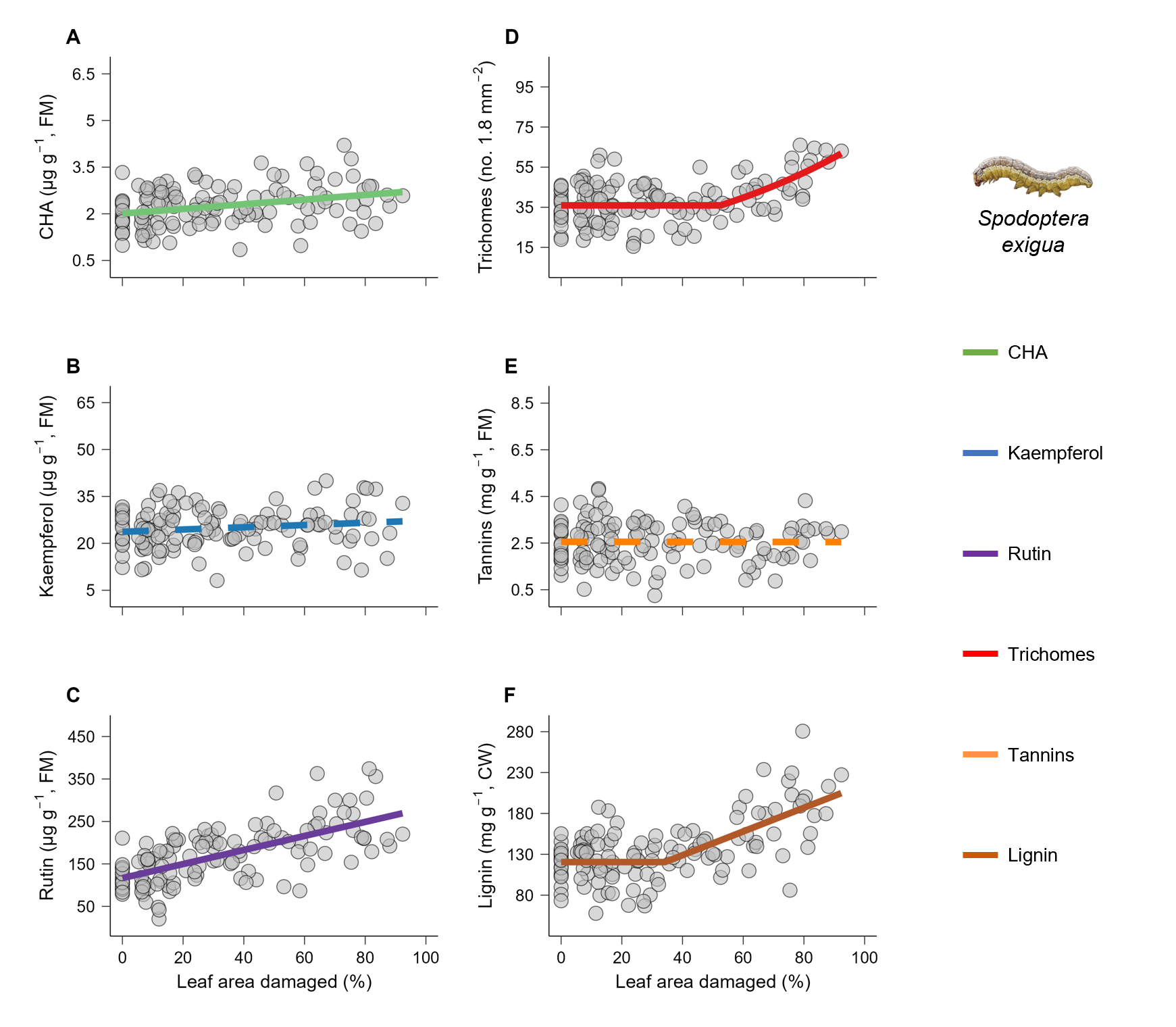

Supplement: S3 Fig — Relationships between levels of each of six traits, chlorogenic acid (CHA), kaempferol, rutin, trichomes, condensed tannins (tannins), and lignin, in Ambrosia artemisiifolia and the percentage of leaf area damaged by S. exigua. Each trait was analyzed separately. The reaction norm for each trait was chosen between a linear or segmented model based on a combination of the Davies test and AIC/QAIC. Data points represent individual replicates (n = 18 per density treatment, seven density treatments). The different line colors represent different traits. Solid lines indicate significant effects (adjusted 95% confidence intervals not crossing zero) of herbivore damage on levels of traits while dotted lines represent nonsignificant effects (adjusted 95% confidence intervals crossing zero). Predicted trichome densities are presented on the original scale (i.e., # per 1.8 mm2). Statistical results are in Table L in S1 File. FM, leaf fresh biomass. CW, cell wall. The data underlying this figure can be found in https://doi.org/10.6084/m9.figshare.29364695. (TIF) [file pbio.3003280.s004.tif]

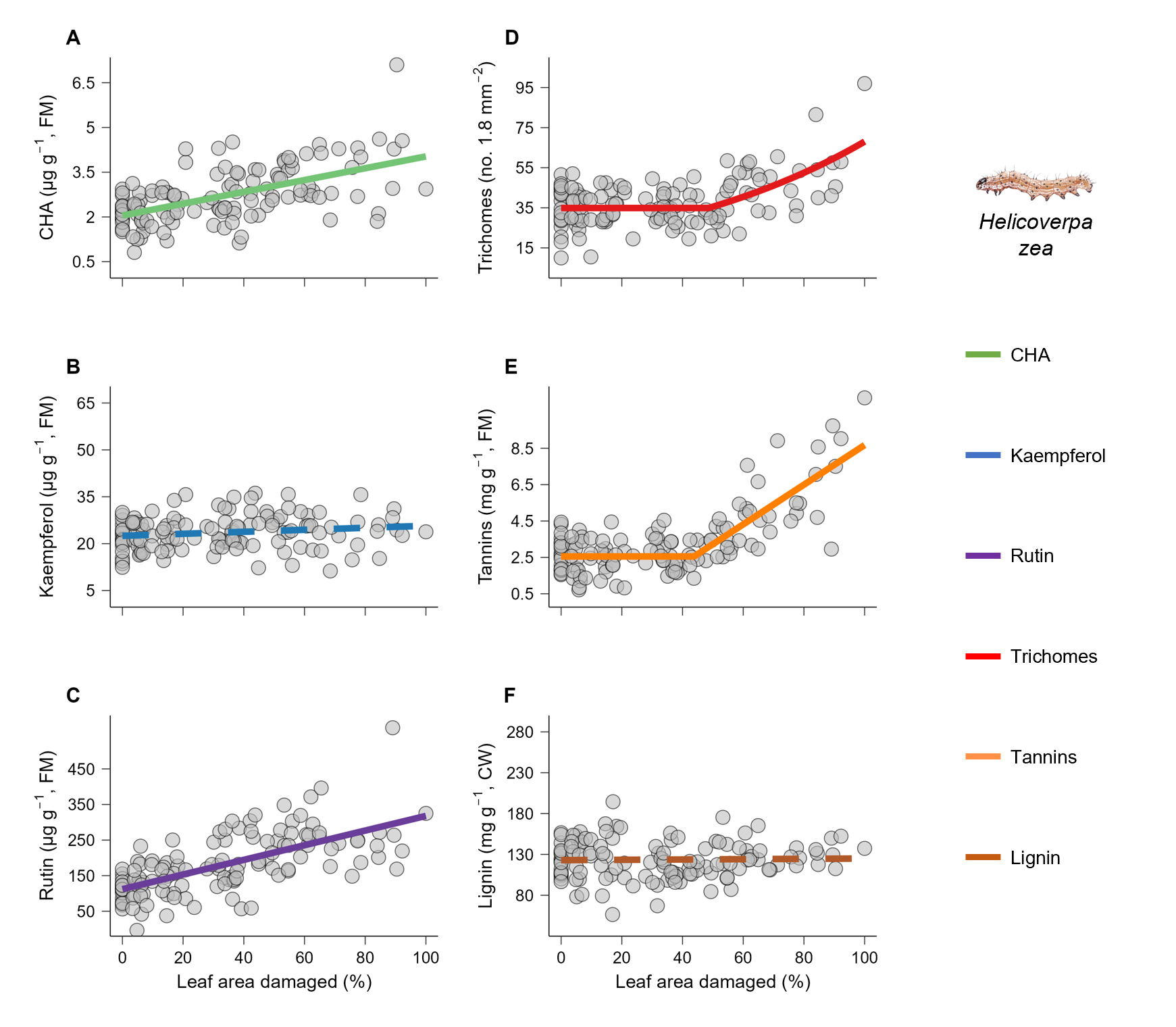

Supplement: S4 Fig — Relationships between levels of each of six traits, chlorogenic acid (CHA), kaempferol, rutin, trichomes, condensed tannins (tannins), and lignin, in Ambrosia artemisiifolia and the percentage of leaf area damaged by H. armigera. Each trait was analyzed separately. The reaction norm for each trait was chosen between a linear or segmented model based on a combination of the Davies test and AIC/QAIC. Data points represent individual replicates (n = 18 per density treatment, seven density treatments). The different line colors represent different traits. Solid lines indicate significant effects (adjusted 95% confidence intervals not crossing zero) of herbivore damage on levels of traits while dotted lines represent nonsignificant effects (adjusted 95% confidence intervals crossing zero). Predicted trichome densities are presented on the original scale (i.e., # per 1.8 mm2). Statistical results are in Table L in S1 File. FM, leaf fresh biomass. CW, cell wall. The data underlying this figure can be found in https://doi.org/10.6084/m9.figshare.29364695. (TIF) [file pbio.3003280.s005.tif]

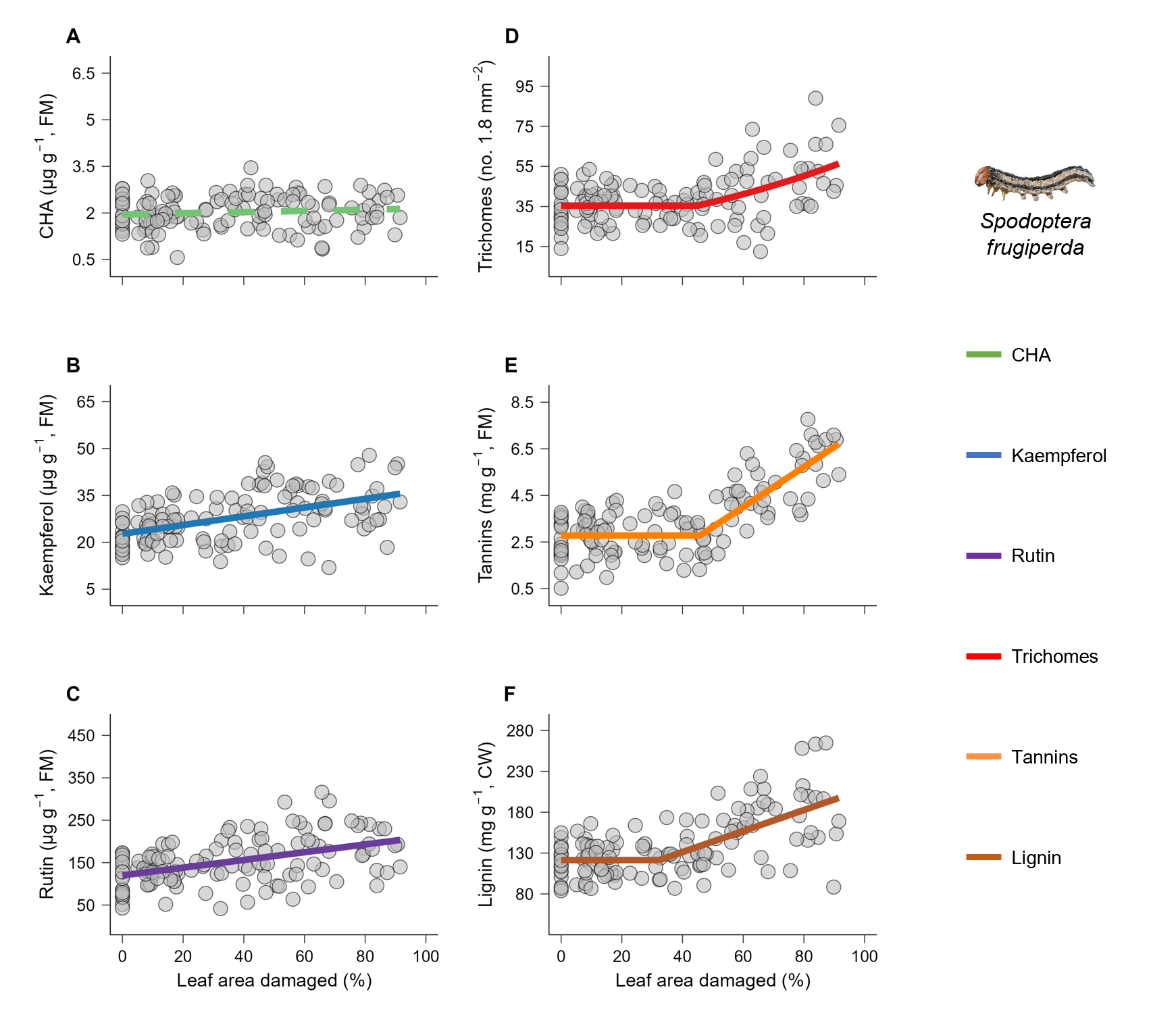

Supplement: S5 Fig — Relationships between levels of each of six traits, chlorogenic acid (CHA), kaempferol, rutin, trichomes, condensed tannins (tannins), and lignin, in Ambrosia artemisiifolia and the percentage of leaf area damaged by S. frugiperda. Each trait was analyzed separately. The reaction norm for each trait was chosen between a linear or segmented model based on a combination of the Davies test and AIC/QAIC. Data points represent individual replicates (n = 18 per density treatment, seven density treatments). The different line colors represent different traits. Solid lines indicate significant effects (adjusted 95% confidence intervals not crossing zero) of herbivore damage on levels of traits while dotted lines represent nonsignificant effects (adjusted 95% confidence intervals crossing zero). Predicted trichome densities are presented on the original scale (i.e., # per 1.8 mm2). Statistical results are in Table L in S1 File. FM, leaf fresh biomass. CW, cell wall. The data underlying this figure can be found in https://doi.org/10.6084/m9.figshare.29364695. (TIF) [file pbio.3003280.s006.tif]

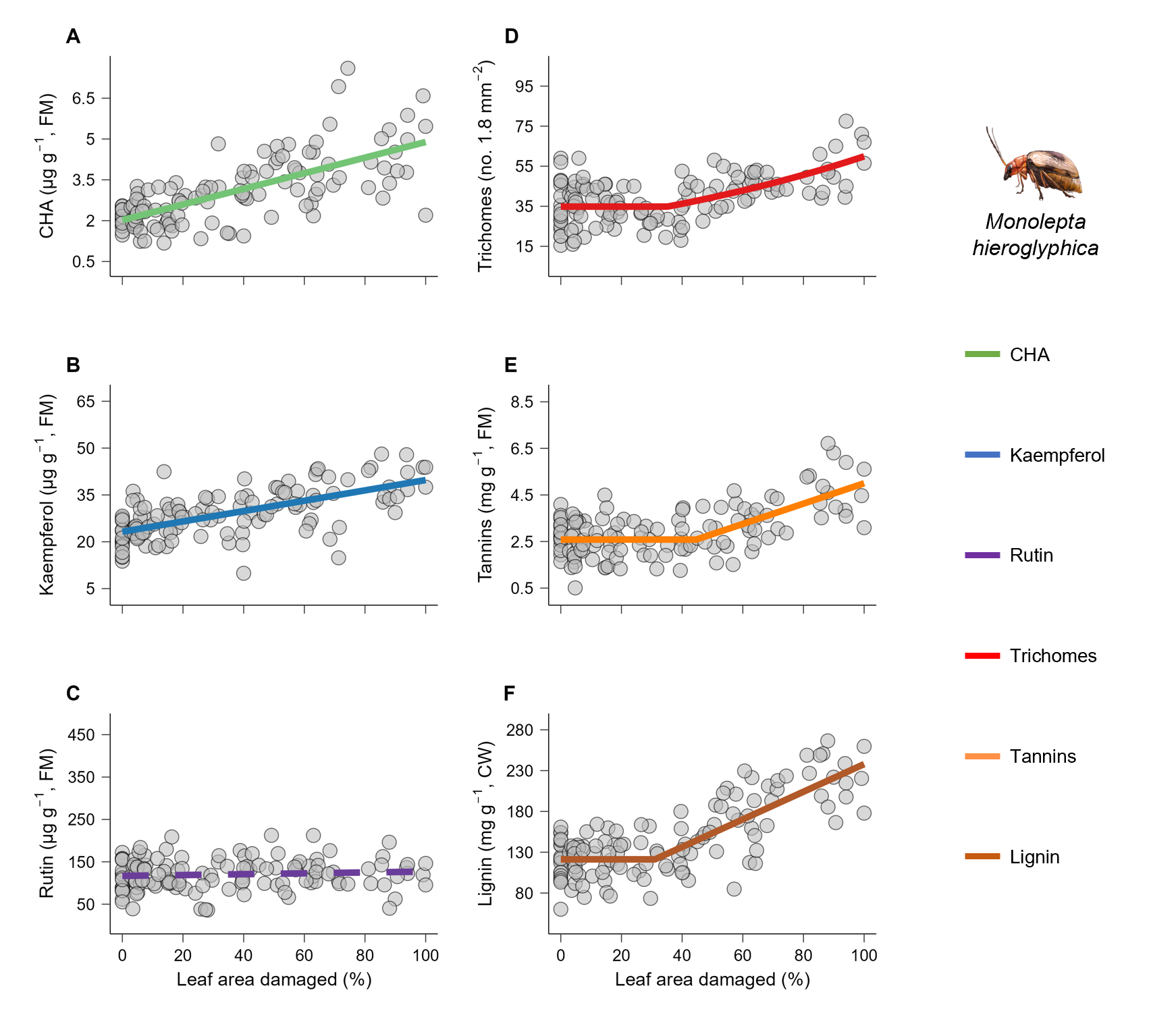

Supplement: S6 Fig — Relationships between levels of each of six traits, chlorogenic acid (CHA), kaempferol, rutin, trichomes, condensed tannins (tannins), and lignin, in Ambrosia artemisiifolia and the percentage of leaf area damaged by M. hieroglyphica. Each trait was analyzed separately. The reaction norm for each trait was chosen between a linear or segmented model based on a combination of the Davies test and AIC/QAIC. Data points represent individual replicates (n = 18 per density treatment, seven density treatments). The different line colors represent different traits. Solid lines indicate significant effects (adjusted 95% confidence intervals not crossing zero) of herbivore damage on levels of traits while dotted lines represent nonsignificant effects (adjusted 95% confidence intervals crossing zero). Predicted trichome densities are presented on the original scale (i.e., # per 1.8 mm2). Statistical results are in Table L in S1 File. FM, leaf fresh biomass. CW, cell wall. The data underlying this figure can be found in https://doi.org/10.6084/m9.figshare.29364695. (TIF) [file pbio.3003280.s007.tif]

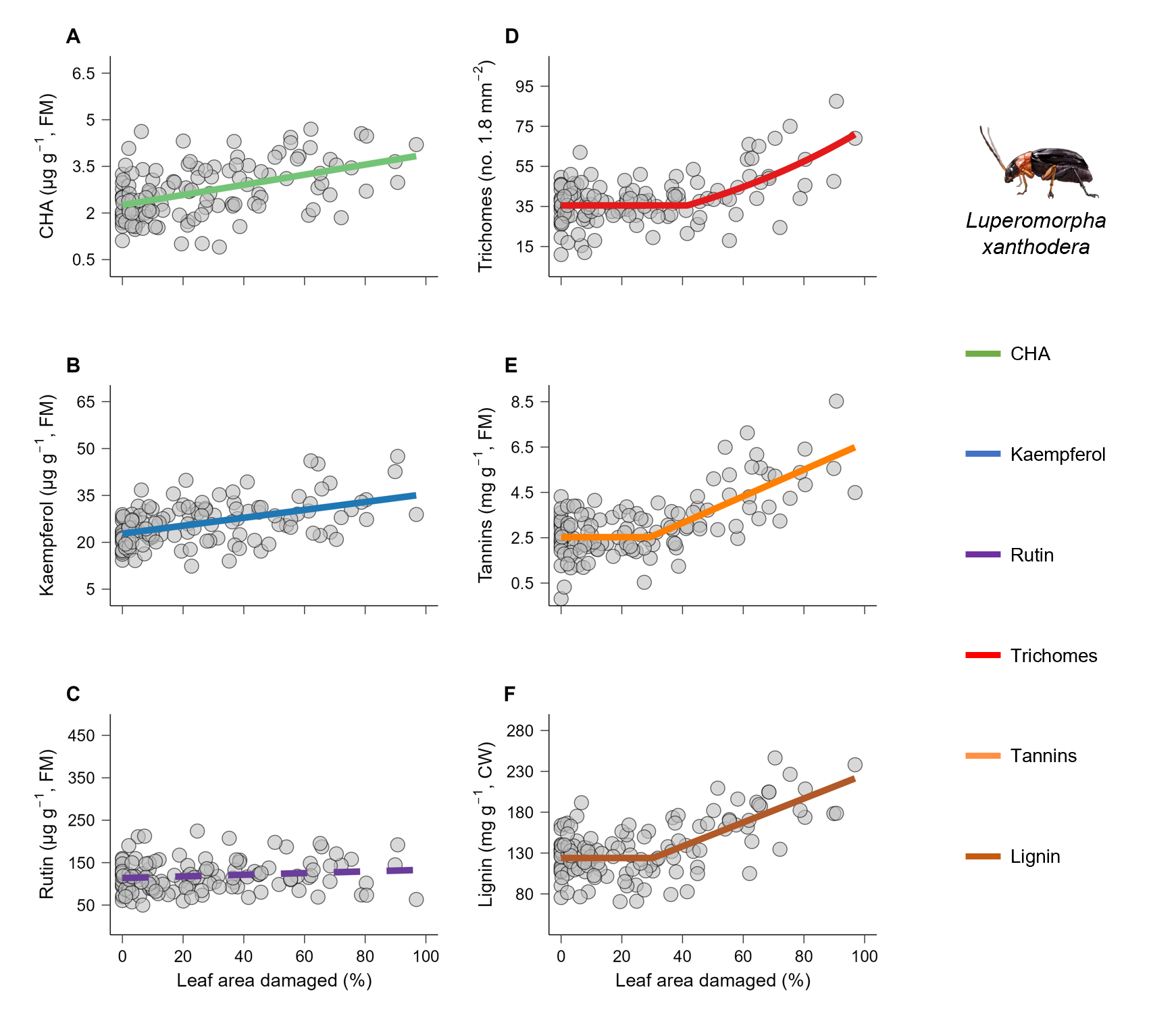

Supplement: S7 Fig — Relationships between levels of each of six traits, chlorogenic acid (CHA), kaempferol, rutin, trichomes, condensed tannins (tannins), and lignin, in Ambrosia artemisiifolia and the percentage of leaf area damaged by L. xanthodera. Each trait was analyzed separately. The reaction norm for each trait was chosen between a linear or segmented model based on a combination of the Davies test and AIC/QAIC. Data points represent individual replicates (n = 18 per density treatment, seven density treatments). The different line colors represent different traits. Solid lines indicate significant effects (adjusted 95% confidence intervals not crossing zero) of herbivore damage on levels of traits while dotted lines represent nonsignificant effects (adjusted 95% confidence intervals crossing zero). Predicted trichome densities are presented on the original scale (i.e., # per 1.8 mm2). Statistical results are in Table L in S1 File. FM, leaf fresh biomass. CW, cell wall. The data underlying this figure can be found in https://doi.org/10.6084/m9.figshare.29364695. (TIF) [file pbio.3003280.s008.tif]

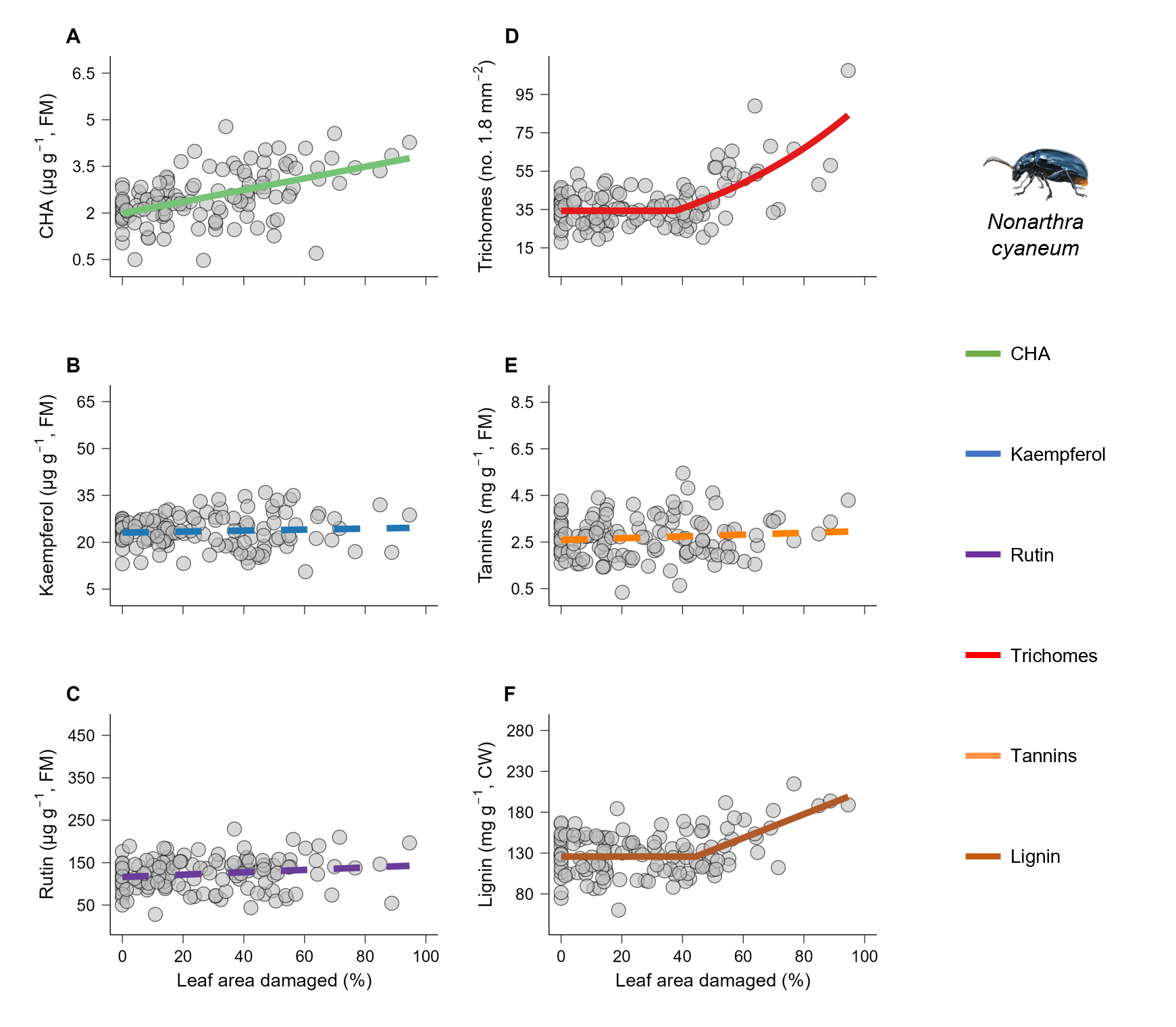

Supplement: S8 Fig — Relationships between levels of each of six traits, chlorogenic acid (CHA), kaempferol, rutin, trichomes, condensed tannins (tannins), and lignin, in Ambrosia artemisiifolia and the percentage of leaf area damaged by N. cyaneum. Each trait was analyzed separately. The reaction norm for each trait was chosen between a linear or segmented model based on a combination of the Davies test and AIC/QAIC. Data points represent individual replicates (n = 18 per density treatment, seven density treatments). The different line colors represent different traits. Solid lines indicate significant effects (adjusted 95% confidence intervals not crossing zero) of herbivore damage on levels of traits while dotted lines represent nonsignificant effects (adjusted 95% confidence intervals crossing zero). Predicted trichome densities are presented on the original scale (i.e., # per 1.8 mm2). Statistical results are in Table L in S1 File. FM, leaf fresh biomass. CW, cell wall. The data underlying this figure can be found in https://doi.org/10.6084/m9.figshare.29364695. (TIF) [file pbio.3003280.s009.tif]

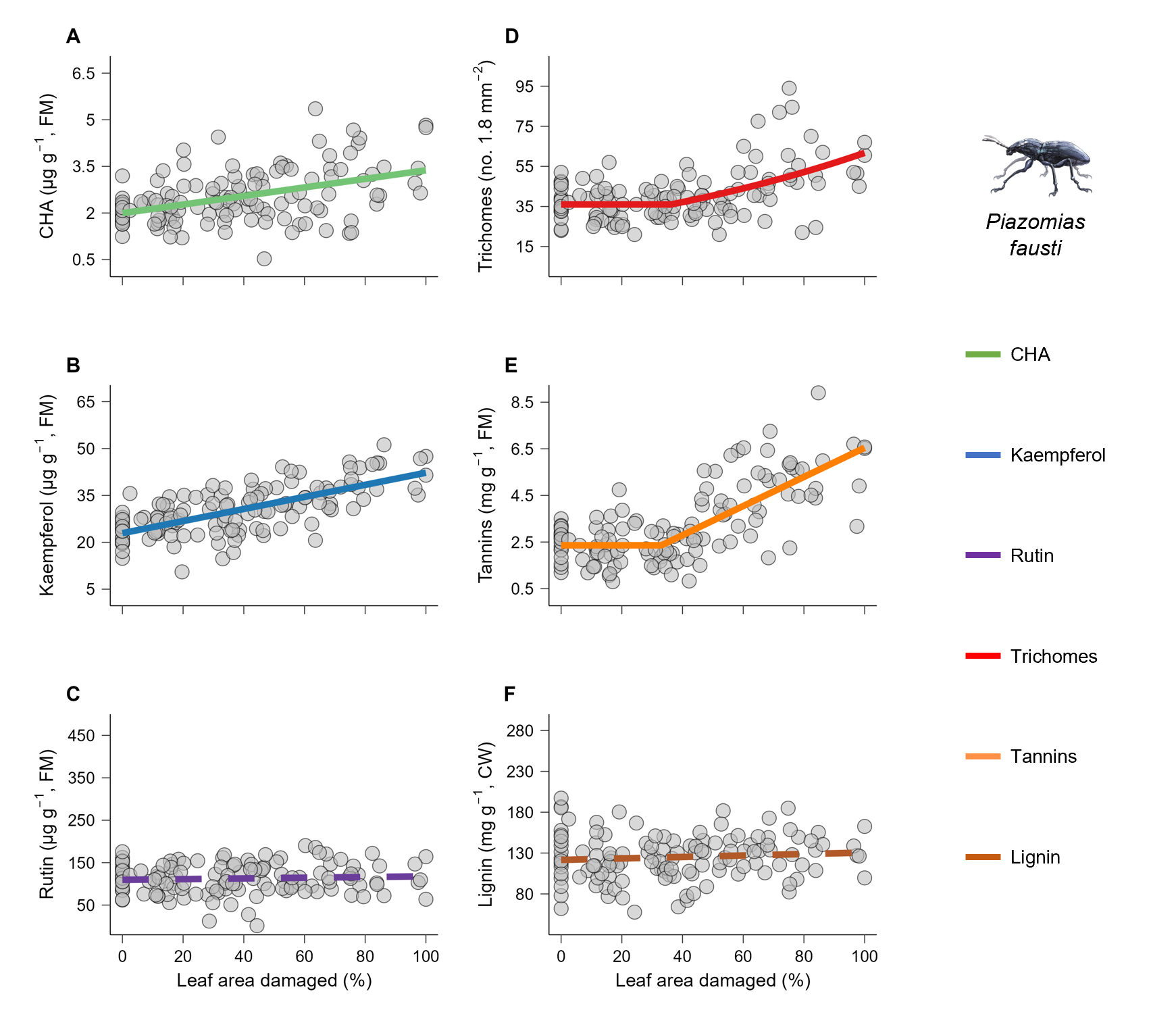

Supplement: S9 Fig — Relationships between levels of each of six traits, chlorogenic acid (CHA), kaempferol, rutin, trichomes, condensed tannins (tannins), and lignin, in Ambrosia artemisiifolia and the percentage of leaf area damaged by P. fausti. Each trait was analyzed separately. The reaction norm for each trait was chosen between a linear or segmented model based on a combination of the Davies test and AIC/QAIC. Data points represent individual replicates (n = 18 per density treatment, seven density treatments). The different line colors represent different traits. Solid lines indicate significant effects (adjusted 95% confidence intervals not crossing zero) of herbivore damage on levels of traits while dotted lines represent nonsignificant effects (adjusted 95% confidence intervals crossing zero). Predicted trichome densities are presented on the original scale (i.e., # per 1.8 mm2). Statistical results are in Table L in S1 File. FM, leaf fresh biomass. CW, cell wall. The data underlying this figure can be found in https://doi.org/10.6084/m9.figshare.29364695. (TIF) [file pbio.3003280.s010.tif]

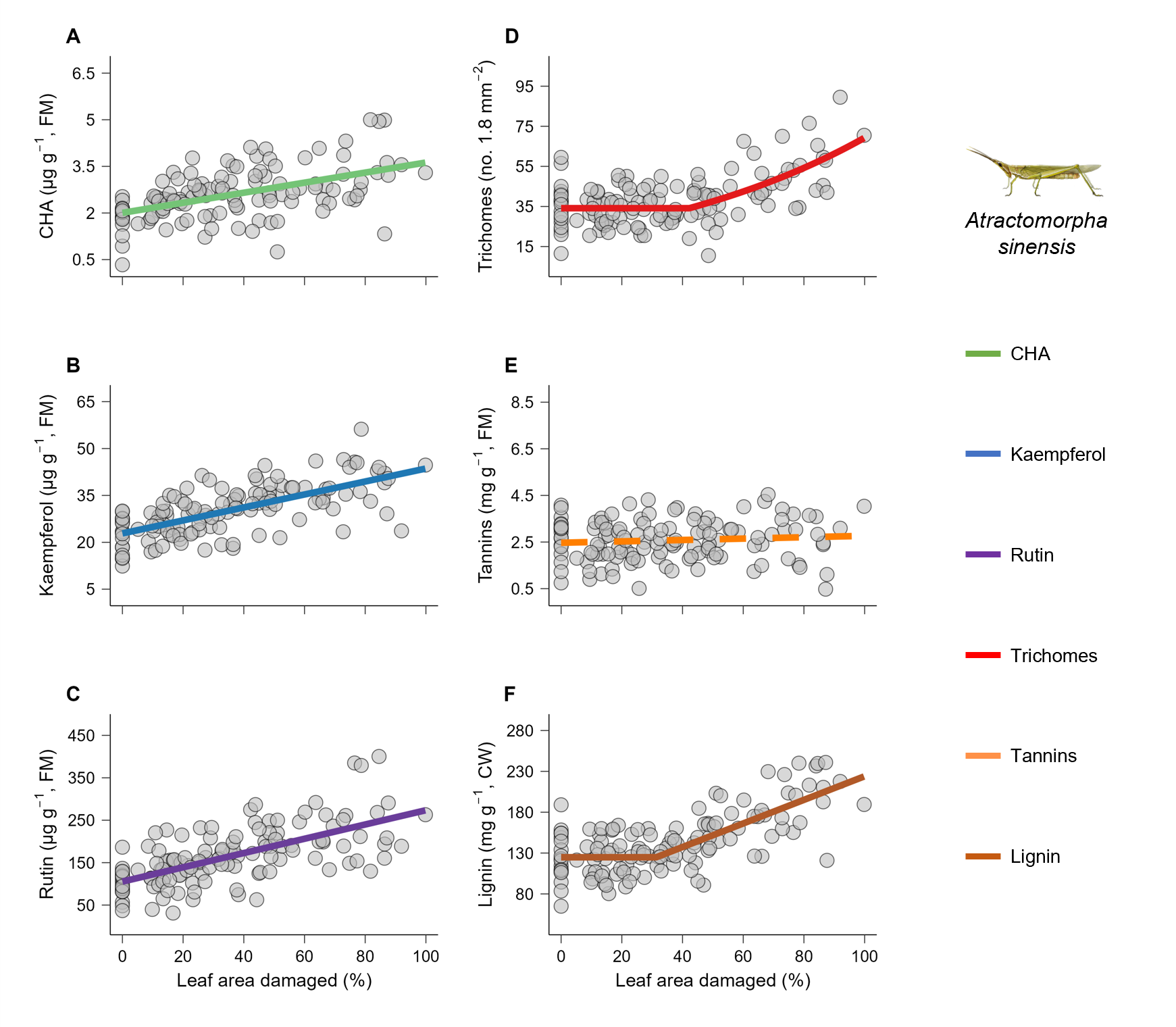

Supplement: S10 Fig — Relationships between levels of each of six traits, chlorogenic acid (CHA), kaempferol, rutin, trichomes, condensed tannins (tannins), and lignin, in Ambrosia artemisiifolia and the percentage of leaf area damaged by A. sinensis. Each trait was analyzed separately. The reaction norm for each trait was chosen between a linear or segmented model based on a combination of the Davies test and AIC/QAIC. Data points represent individual replicates (n = 18 per density treatment, seven density treatments). The different line colors represent different traits. Solid lines indicate significant effects (adjusted 95% confidence intervals not crossing zero) of herbivore damage on levels of traits while dotted lines represent nonsignificant effects (adjusted 95% confidence intervals crossing zero). Predicted trichome densities are presented on the original scale (i.e., # per 1.8 mm2). Statistical results are in Table L in S1 File. FM, leaf fresh biomass. CW, cell wall. The data underlying this figure can be found in https://doi.org/10.6084/m9.figshare.29364695. (TIF) [file pbio.3003280.s011.tif]

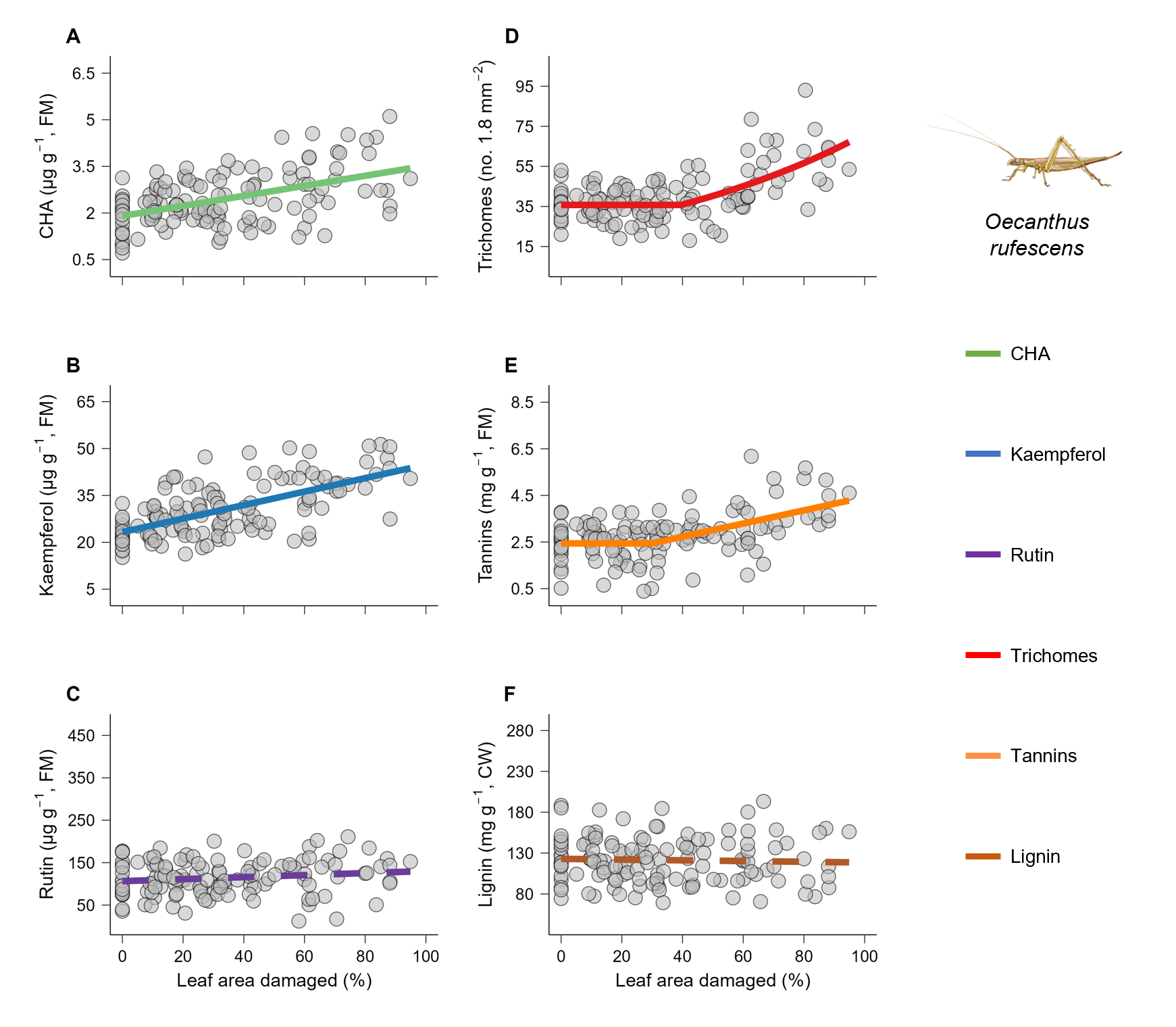

Supplement: S11 Fig — Relationships between levels of each of six traits, chlorogenic acid (CHA), kaempferol, rutin, trichomes, condensed tannins (tannins), and lignin, in Ambrosia artemisiifolia and the percentage of leaf area damaged by O. rufescens. Each trait was analyzed separately. The reaction norm for each trait was chosen between a linear or segmented model based on a combination of the Davies test and AIC/QAIC. Data points represent individual replicates (n = 18 per density treatment, seven density treatments). The different line colors represent different traits. Solid lines indicate significant effects (adjusted 95% confidence intervals not crossing zero) of herbivore damage on levels of traits while dotted lines represent nonsignificant effects (adjusted 95% confidence intervals crossing zero). Predicted trichome densities are presented on the original scale (i.e., # per 1.8 mm2). Statistical results are in Table L in S1 File. FM, leaf fresh biomass. CW, cell wall. The data underlying this figure can be found in https://doi.org/10.6084/m9.figshare.29364695. (TIF) [file pbio.3003280.s012.tif]

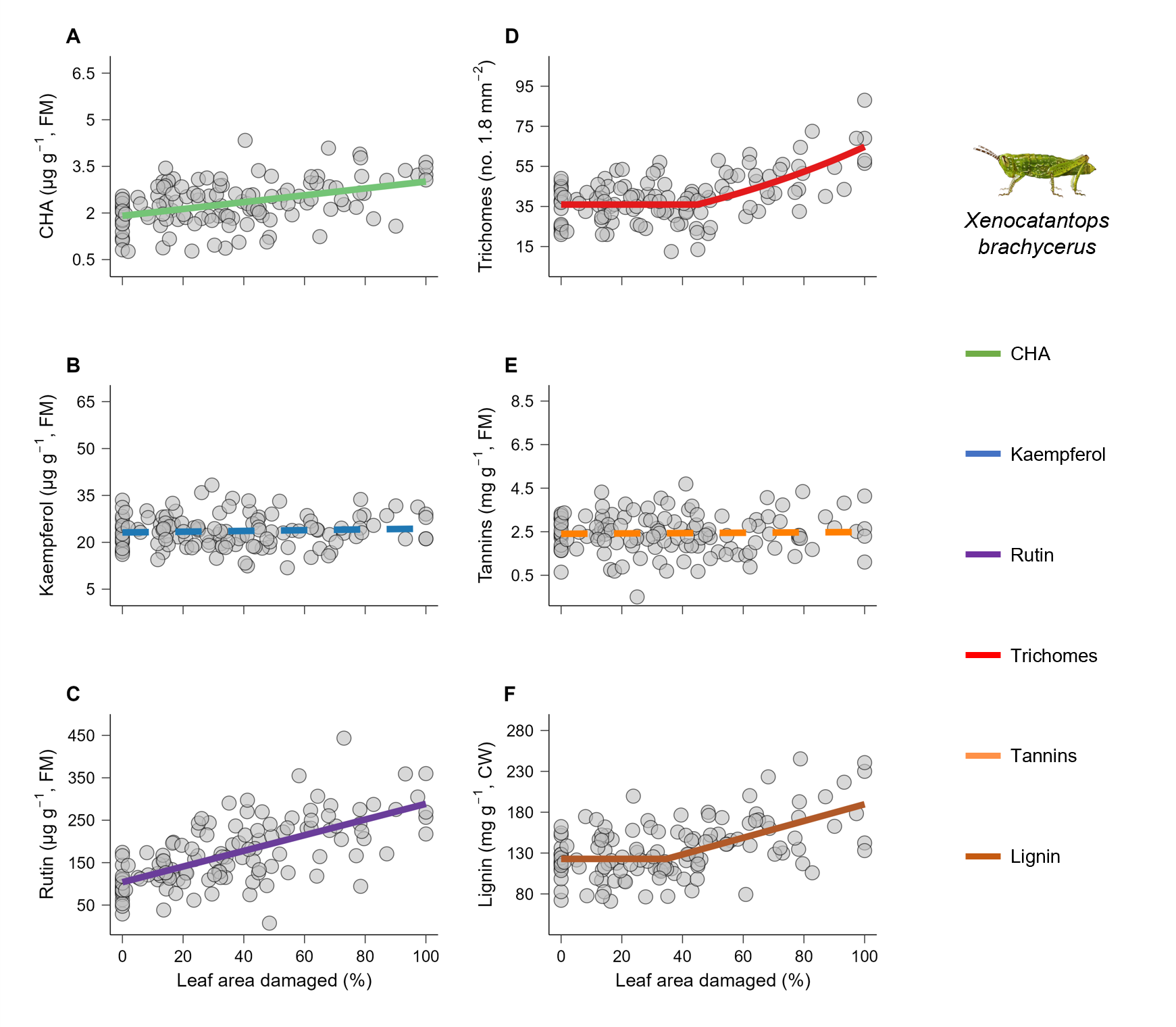

Supplement: S12 Fig — Relationships between levels of each of six traits, chlorogenic acid (CHA), kaempferol, rutin, trichomes, condensed tannins (tannins), and lignin, in Ambrosia artemisiifolia and the percentage of leaf area damaged by X. brachycerus. Each trait was analyzed separately. The reaction norm for each trait was chosen between a linear or segmented model based on a combination of the Davies test and AIC/QAIC. Data points represent individual replicates (n = 18 per density treatment, seven density treatments). The different line colors represent different traits. Solid lines indicate significant effects (adjusted 95% confidence intervals not crossing zero) of herbivore damage on levels of traits while dotted lines represent nonsignificant effects (adjusted 95% confidence intervals crossing zero). Predicted trichome densities are presented on the original scale (i.e., # per 1.8 mm2). Statistical results are in Table L in S1 File. FM, leaf fresh biomass. CW, cell wall. The data underlying this figure can be found in https://doi.org/10.6084/m9.figshare.29364695. (TIF) [file pbio.3003280.s013.tif]

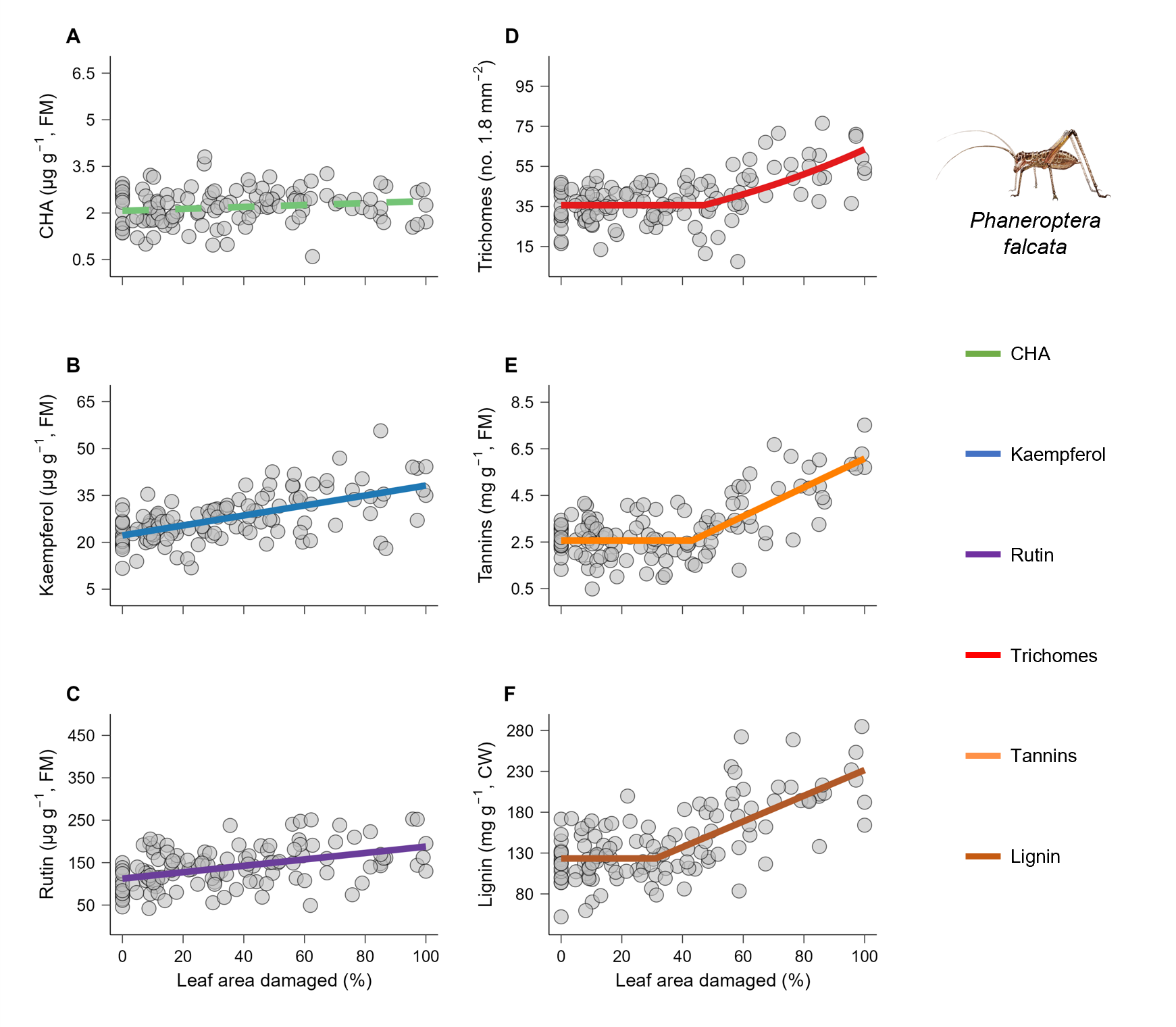

Supplement: S13 Fig — Relationships between levels of each of six traits, chlorogenic acid (CHA), kaempferol, rutin, trichomes, condensed tannins (tannins), and lignin, in Ambrosia artemisiifolia and the percentage of leaf area damaged by P. falcata. Each trait was analyzed separately. The reaction norm for each trait was chosen between a linear or segmented model based on a combination of the Davies test and AIC/QAIC. Data points represent individual replicates (n = 18 per density treatment, seven density treatments). The different line colors represent different traits. Solid lines indicate significant effects (adjusted 95% confidence intervals not crossing zero) of herbivore damage on levels of traits while dotted lines represent nonsignificant effects (adjusted 95% confidence intervals crossing zero). Predicted trichome densities are presented on the original scale (i.e., # per 1.8 mm2). Statistical results are in Table L in S1 File. FM, leaf fresh biomass. CW, cell wall. The data underlying this figure can be found in https://doi.org/10.6084/m9.figshare.29364695. (TIF) [file pbio.3003280.s014.tif]
